# Integrated multi-omics deciphers sepsis immune dysregulation: a dual-pathway targeted small-molecule therapy improves survival and ameliorates multi-organ dysfunction

**DOI:** 10.3389/fimmu.2026.1809540

**Published:** 2026-05-15

**Authors:** Jiawen Duan, Lingyu Jiang, Kunlin Hu, Huirong Shi, Xinyu Chi, Weiting Feng, Guodong Wang, Shulin Xiang, Bin Xiong

**Affiliations:** 1 Guangxi Health Commission Key Laboratory of Diagnosis and Treatment of Acute Respiratory Distress Syndrome, Guangxi Academy of Medical Sciences, Nanning, Guangxi, China; 2 Institute of Infectious Diseases and Emergency and Critical Care Medicine, Guangxi Academy of Medical Sciences, Nanning, Guangxi, China; 3 Department of Intensive Care Unit, The People’s Hospital of Guangxi Zhuang Autonomous Region, Nanning, Guangxi, China; 4 Graduate School, Youjiang Medical University for Nationalities, Baise, Guangxi, China; 5 Guangxi Zhuang Autonomous Region Engineering Research Center for 3D Printing in Smart Biomanufacturing and Application, Guangxi Academy of Medical Sciences, Nanning, Guangxi, China; 6 Guangxi Clinical Research Center for Critical Treatment of Major Infectious Diseases, Nanning, Guangxi, China

**Keywords:** immune dysregulation, multi-omics integration, sepsis, small-molecule combination therapy, therapeutic targets

## Abstract

**Introduction:**

Sepsis is a life-threatening organ dysfunction syndrome with persistently high global mortality, driven by dysregulated host immune response. Existing single-target therapies fail to simultaneously address hyperinflammation and impaired tissue repair, leading to limited clinical efficacy and repeated translational failures.

**Methods:**

Integrated multi-omics datasets (scRNA-seq, miRNA-seq, blood/lung RNA-seq) delineated immune cell dynamics and dysregulated pathways in sepsis. Guided by omics findings, we designed two small-molecule combinations (C1, C2) targeting the identified pathways. Their efficacy and mechanism were validated in *in vitro* and *in vivo* sepsis models.

**Results:**

Septic patients showed a hallmark immune remodeling signature: expansion of pro-inflammatory myeloid cells (neutrophils, monocytes) and depletion of protective lymphoid cells (B cells, NK cells). A dual-pathway small-molecule combination C2 targeting inflammatory cascades and Hippo/Wnt regenerative pathways, exerted significant synergistic therapeutic effects. It robustly rebalanced systemic and organ-specific inflammation (suppressed *Il1b*, *Il6*, *Nos2*, *Tnfa*; elevated *Il10*, *Arg1*, *Tgfb*, *Nos3*) *in vitro* and *in vivo*, ameliorated multi-organ injury, and improved 7-day survival in septic mice from 20% (untreated) to 70%. Single-compound control experiments confirmed that the enhanced efficacy of C2 stems from dual-pathway synergy, not individual components.

**Discussion:**

This study revealed immune dysregulation as the core pathogenesis of sepsis. The C2 combination simultaneously mitigates hyperinflammation and promotes tissue repair, providing a novel, mechanism-driven, and clinically translatable combination strategy for sepsis management.

## Graphical Abstract

**Figure:**
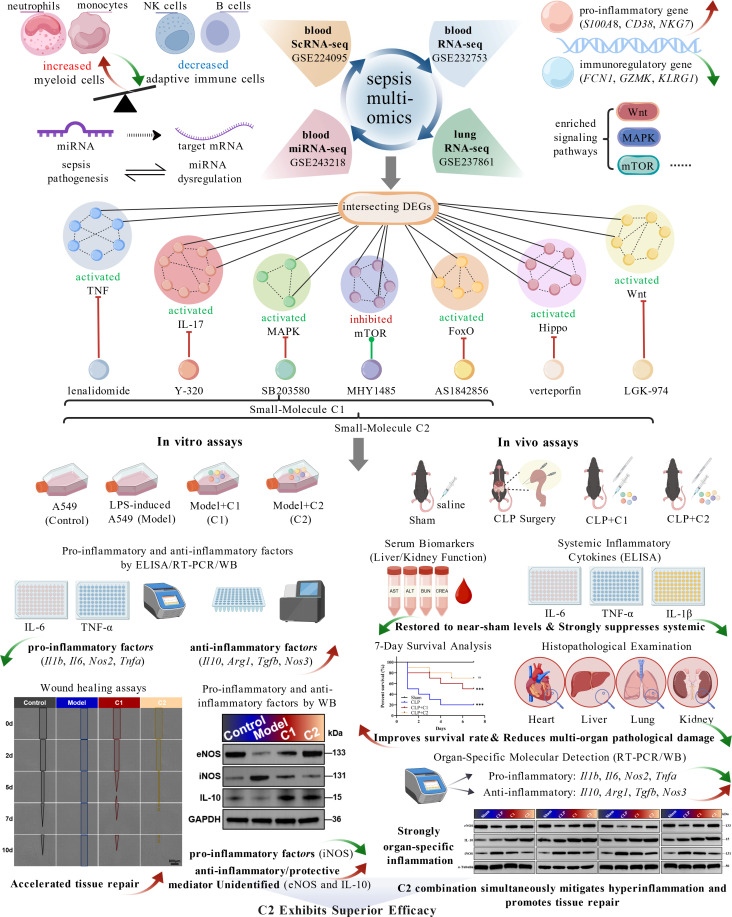

## 1 Introduction

Sepsis, defined as life-threatening organ dysfunction resulting from a dysregulated host response to infection, remains a devastating challenge in critical care medicine. It accounts for nearly 20% of all fatalities worldwide, representing the leading cause of mortality in intensive care units (ICUs) (1, 2). Despite decades of advancements in supportive care, antimicrobial therapy, and resuscitation strategies, sepsis mortality rates remain persistently high at 30–50% (3, 4). This burden is exacerbated by an aging population, increasing antibiotic resistance, and the inability of conventional treatments to target the underlying immunopathological cascade of sepsis (5).

The core pathogenesis of sepsis lies in profound and systemic immune dysregulation. An initial “cytokine storm”, driven by hyperactivated innate immunity, is followed by a state of immunoparalysis. This immunosuppressive state is characterized by impaired adaptive immunity, defective pathogen clearance (6, 7), and sustained expansion of immunosuppressive cell populations (e.g., myeloid-derived suppressor cells, regulatory T cells) (8). This paradoxical imbalance between hyperinflammation and immunosuppression drives the development of multiple organ dysfunction syndrome (MODS), the primary cause of sepsis-related death. These findings underscore the urgent need for targeted therapies that restore immune homeostasis, rather than merely suppressing inflammation (9, 10).

Over the past decade, high-throughput omics technologies have advanced our understanding of complex diseases by capturing cellular, molecular, and regulatory heterogeneity at an unprecedented scale (11, 12). Single-cell RNA sequencing (scRNA-seq) has enabled high-resolution dissection of immune cell dynamics in sepsis, revealing lineage-specific alterations in gene expression and cell population proportions that were obscured by bulk sequencing (13, 14). Complementary to scRNA-seq, miRNA sequencing (miRNA-seq) reveals post-transcriptional regulatory networks. miRNAs act as key modulators of immune cell activation, inflammation, and apoptosis by repressing target mRNA translation or promoting degradation (15, 16). Bulk RNA sequencing of blood and target tissues (the primary site of sepsis-induced injury) further validated tissue-specific transcriptional changes and reinforced consensus dysregulated pathways (17, 18). Integrating these multi-omics datasets (scRNA-seq, miRNA-seq, bulk RNA-seq) provides a holistic view of sepsis pathogenesis. This approach enables the identification of core molecular networks, key signaling pathways, and potential therapeutic targets that may be missed by single-omics approaches—a strategy increasingly validated by recent precision medicine studies (19, 20).

Small-molecule combinations represent a promising class of therapeutics for sepsis. This is due to their favorable pharmacokinetic properties (e.g., membrane permeability, tissue bioavailability) and their ability to be rationally designed to target specific signaling pathways or molecular nodes (21, 22). Unlike single-target drugs, which often fail to address the multifactorial nature of sepsis, combination therapies can synergistically modulate multiple dysregulated pathways, simultaneously mitigating hyperinflammation and promoting tissue repair (23, 24). Recent preclinical and clinical studies support this approach: combinations involving melatonin, metformin, or herbal extracts have shown efficacy in reducing organ damage and improving survival by targeting inflammation, oxidative stress, and metabolic reprogramming (19, 25). However, the development of effective small-molecule combinations for sepsis has been hindered by a lack of systematic, omics-driven target identification. Prior strategies often relied on hypothesis-driven targeting of individual inflammatory pathways, without accounting for the interconnectedness of immune regulation and tissue regeneration.

To date, clinical management of sepsis remains dominated by supportive care, and the development of immunomodulatory therapies has long been plagued by translational bottlenecks. Existing immunomodulatory therapies for sepsis primarily fall into two single-direction intervention categories, both with inherent limitations that hinder clinical efficacy. The first category, anti-inflammatory immunotherapies, includes three main types of single-target agents: glucocorticoids, endotoxin-targeted therapies, and specific pro-inflammatory mediator inhibitors. High-dose glucocorticoids significantly increase mortality, while low physiological doses only improve hemodynamic stability without providing survival benefits (26). Endotoxin-targeted therapies have shown no consistent survival advantage in 10 clinical trials (27). Specific pro-inflammatory mediator inhibitors (including anti-TNF-α agents, IL-1 receptor antagonists) failed to show consistent survival benefits in phase III clinical trials, with high-dose regimens even increasing mortality risk (28). The second category, immune-activating therapies developed for sepsis-induced immunosuppression, includes immune-stimulating cytokines (IFN-γ, GM-CSF, IL-7), immune checkpoint inhibitors (anti-PD-1/PD-L1, anti-TIM-3), thymosin α1, and intravenous immunoglobulin. These agents only partially restore innate/adaptive immune cell function, but fail to alleviate early hyperinflammation or established organ damage (29, 30). Collectively, existing therapies adopt a single-target or single-direction intervention mode that cannot simultaneously address the two core pathological processes of sepsis: excessive inflammatory response and organ injury/impaired repair, leading to markedly restricted clinical efficacy and repeated translational failures (31, 32). Thus, there is an urgent clinical need for a novel immunomodulatory strategy that can simultaneously normalize dysregulated immune responses and promote repair of injured organs in sepsis.

In this study, we systematically investigated the immune dysregulation landscape of sepsis via integrated multi-omics analysis of four independent datasets, and identified core dysregulated pathways driving both hyperinflammation and organ injury in sepsis. Based on these findings, we developed a novel dual-pathway small-molecule combination therapy, which not only targets multiple classical inflammatory pathways (TNF, IL-17, MAPK, mTOR, FoxO) to suppress excessive inflammatory responses but also modulates Hippo/Wnt signaling to reverse sepsis-induced organ damage. This strategy addresses a key shortcoming of existing single-target anti-inflammatory therapies, which focus only on inflammation inhibition without organ protection, and achieves synergistic regulation of immune homeostasis and tissue repair. We further validated the therapeutic efficacy of this strategy in both *in vitro* sepsis cell model and *in vivo* cecal ligation and puncture (CLP) mouse model, to provide a novel and translational therapeutic option for clinical sepsis management.

## 2 Methods

### 2.1 Multi-omics data processing and integrated bioinformatic analysis

#### 2.1.1 Public multi-omics datasets acquisition

All human sepsis-related omics datasets were retrieved from the Gene Expression Omnibus (GEO) database, with strict matching of sample type and disease phenotype. Four complementary omics layers were included: peripheral blood scRNA-seq data (GSE224095, DOI: 10.2478/aspr-2023-0030) from 5 septic patients and 5 healthy controls, which were used to delineate immune cell subset dynamics and gene expression alterations; whole blood miRNA-seq data (GSE243218, DOI: 10.1186/s12985-024-02451-6) from 22 septic patients and 15 healthy subjects, for identifying dysregulated post-transcriptional regulators and target mRNAs; peripheral blood bulk RNA-seq data (GSE232753, DOI: 10.3390/ijms24119362) to validate systemic transcriptional dysregulation; and lung tissue bulk RNA-seq data (GSE237861, DOI: 10.1111/jcmm.17938) to characterize tissue-specific transcriptional changes in sepsis-induced organ injury.

#### 2.1.2 Single-cell RNA-seq data processing and analysis

All the scRNA-seq analyses were performed in R (v4.2.3) using the Seurat R package (v4.3.0). Raw gene-barcode matrices were converted into a Seurat object for quality control (QC), with low-quality cells (gene counts <200 or >2500, mitochondrial gene ratio >10%) and potential doublets filtered out. The filtered matrix was normalized via the LogNormalize method (scale factor = 10,000), and the top 2000 highly variable genes (HVGs) were identified using the “vst” method. Principal component analysis (PCA) was performed on the scaled HVG matrix, and the top 9 PCs (PCs 0 to 8) were selected based on the elbow plot for subsequent non-linear dimensionality reduction and clustering. Uniform Manifold Approximation and Projection (UMAP) and t-distributed Stochastic Neighbor Embedding (tSNE, perplexity = 30) were applied for data visualization. Unsupervised cell clustering was conducted via the graph-based Louvain algorithm with a resolution of 0.1, yielding 9 distinct clusters. These clusters were annotated using the SingleR R package (v2.2.0) with the Human Primary Cell Atlas (HPCA) as the reference dataset, and further validated by canonical marker genes. The relative proportion of each cell type in healthy controls and septic patients was calculated via the prop.table function, and visualized as a stacked bar plot using ggplot2 R package (v3.4.2). Cluster-specific marker genes were identified using the Wilcoxon rank-sum test (FindAllMarkers function), and their spatial expression distribution was visualized via FeaturePlot on UMAP and tSNE embeddings.

#### 2.1.3 miRNA-seq data processing and target gene prediction

miRNA-seq analyses were performed in R (v4.2.3). Raw count data were normalized via quantile normalization in the LIMMA R package (v3.54.2). Sample consistency was validated using PCA (stats R package v3.6.2), UMAP (umap R package v0.2.10.0), and tSNE (Rtsne R package v0.16). Differentially expressed miRNAs between sepsis and control groups were identified via LIMMA with thresholds of raw *p*-value < 0.05 and |log_2_(fold change, FC)| ≥ 1.5. These differentially expressed miRNAs were visualized as volcano plots (ggplot2 v3.4.2) and hierarchical clustering heatmaps (pheatmap R package v1.0.12) after log_2_-transformation and z-score scaling. High-confidence target mRNAs of dysregulated miRNAs were predicted using TargetScan (v7.2, https://www.targetscan.org/vert_72/) and miRDB (v6.0, https://mirdb.org/mirdb/index.html). Only target genes identified by both databases were retained for subsequent integration analysis; Venn diagrams (VennDiagram R package v1.7.3) were used to visualize the overlapping target genes.

#### 2.1.4 Bulk RNA-seq data processing and functional enrichment analysis

Whole blood and lung tissue bulk RNA-seq data were processed in R (v4.2.3) with a unified workflow. Raw read counts were preprocessed via voom transformation and quantile normalization in LIMMA. Data quality was verified via PCA, UMAP, tSNE, and sample correlation heatmaps (pheatmap v1.0.12). Differentially expressed mRNAs (DEGs) were identified using LIMMA with thresholds of adjusted *p*-value < 0.05 and |log_2_FC| ≥ 1.5, visualized via volcano plots and heatmaps. Gene Ontology (GO) and Kyoto Encyclopedia of Genes and Genomes (KEGG) enrichment analyses were performed using clusterProfiler R package (v4.6.2), covering Biological Process (BP), Cellular Component (CC), and Molecular Function (MF) domains. Significantly enriched terms defined by an adjusted *p*-value < 0.05, and visualized as bubble plots and bar plots (ggplot2 v3.4.2).

#### 2.1.5 Multi-omics integration analytical workflow

Multi-omics integration analyses were performed in R (v4.2.3) with standardized pipelines. Prior to integration, gene identifiers across all datasets were standardized to HGNC-approved symbols using org.Hs.eg.db R package (v3.16.0), with unannotated or mismatched genes excluded. Cross-omics differential gene set intersection analysis was performed on four gene sets: scRNA-seq DEGs (adjusted *p*-value < 0.05, |log_2_FC| ≥ 1.0), miRNA-seq high-confidence target mRNAs, blood bulk RNA-seq DEGs (adjusted *p*-value < 0.05, |log_2_FC| ≥ 1.5), and lung bulk RNA-seq DEGs (adjusted *p*-value < 0.05, |log_2_FC| ≥ 1.5). Genes overlapping across all four datasets were defined as consensus sepsis-associated dysregulated genes and subjected to functional enrichment. For miRNA-mRNA regulatory axis integration, dysregulated miRNA target mRNAs were intersected with transcriptomic DEGs, retaining only pairs where both miRNA and mRNA were significantly dysregulated. Systemic and tissue-specific transcriptomic integration was performed by separating DEGs into shared (dysregulated in both blood and lung) and lung-specific subsets, with separate enrichment analyses. Cell phenotype-pathway association mapping linked enriched KEGG pathways to scRNA-seq-defined immune cell subsets, quantifying pathway gene enrichment in each cell type to identify populations driving pathological pathway dysregulation.

#### 2.1.6 Pathway prioritization and therapeutic target selection pipeline

To systematically prioritize the core dysregulated signaling pathways for rational small-molecule therapy design, we established a multi-layered, evidence-based pathway ranking and filtering workflow, which integrated statistical significance, cross-omics consistency, biological relevance, and druggability. All analyses were performed in R (v4.2.3) with pre-specified, objective criteria at each step, as detailed below:

1. Primary pathway screening via cross-omics enrichment consistency.

First, we collected all significantly enriched KEGG pathways from each individual omics dataset, including (1): pathways enriched by DEGs from scRNA-seq (2); pathways enriched by high-confidence target mRNAs of dysregulated miRNAs from miRNA-seq (3); pathways enriched by DEGs from whole blood bulk RNA-seq; (4) pathways enriched by DEGs from lung tissue bulk RNA-seq. The significance threshold for enriched pathways in each dataset was pre-defined as adjusted *p*-value < 0.05. Only pathways significantly enriched in ≥3 out of the 4 omics layers were retained as high-priority candidate pathways. This cross-omics consistency filter ensured the selected pathways reflected universal and robust dysregulation in sepsis, rather than dataset-specific or platform-specific bias.

2. Secondary filtering based on pathophysiological relevance to sepsis.

For the candidate pathways retained from the primary screening, we performed a systematic, literature-based curation to evaluate their established causal roles in the core pathophysiology of sepsis. We focused on four key functional domains: (1) regulation of innate/adaptive immune dysregulation; (2) mediation of hyperinflammatory response and cytokine storm; (3) modulation of tissue repair and sepsis-induced organ injury; (4) involvement in immune cell activation, polarization, and apoptosis. Pathways with well-documented causal roles in sepsis progression, rather than merely correlative expression changes, were prioritized for downstream analysis.

3. Tertiary ranking by magnitude of dysregulation and statistical significance.

We further ranked the retained pathways using two pre-defined quantitative metrics: (1) the adjusted *p*-value of enrichment in the consensus sepsis-associated dysregulated gene set (from the four-omics intersection analysis); (2) the gene ratio (defined as the number of dysregulated genes in the pathway divided by the total number of background genes in the pathway) from the enrichment analysis. Pathways with lower adjusted *p*-values and higher gene ratios were assigned higher priority, as these metrics reflected the extent and statistical robustness of pathway dysregulation in sepsis.

4. Final target pathway selection based on druggability and translational potential.

For the top-ranked pathways, we evaluated their druggability via the DrugBank database and peer-reviewed preclinical literature. This step confirmed the availability of well-characterized, selective small-molecule modulators (inhibitors or activators) with established *in vitro* and *in vivo* safety profiles.

Based on this standardized workflow, we prioritized two categories of core pathways for therapeutic design: (1) classical inflammatory regulatory axes, including the TNF, IL-17, MAPK, mTOR, and FoxO signaling pathways. These pathways were consistently enriched across all four omics datasets and had well-established causal roles in driving sepsis-induced hyperinflammation and systemic immune dysregulation; (2) regenerative and organ-protective pathways, including the Hippo and Wnt signaling pathways. These pathways were significantly dysregulated in both circulating blood and injured lung tissue, and have been validated to mediate sepsis-induced tissue damage and impaired endogenous repair. This category addresses the unmet need of conventional anti-inflammatory-only therapies for sepsis.

### 2.2 Sepsis model establishment and treatment

Human alveolar epithelial A549 cells (Control group) were cultured in Dulbecco’s Modified Eagle’s Medium (DMEM, Cat. #11965-092, Gibco) supplemented with 10% fetal bovine serum (FBS, Cat. #10099-141, Gibco), 1% penicillin-streptomycin (Cat. #15140122, Gibco), and 2 mM L-glutamine (Cat. #25030081, Gibco). The cells were maintained at 37 C in a humidified 5% CO_2_ incubator. To simulate the inflammatory response associated with sepsis, an *in vitro* cell model was established by stimulating A549 cells (Model group) with 1 µg/mL lipopolysaccharide (LPS, Escherichia coli O111:B4, Cat. #L2630, Sigma-Aldrich) for 24h. Two screened small-molecule combinations (C1 and C2 group) were added to the sepsis cell models separately to evaluate their therapeutic effects on sepsis. Combination 1 (C1) consists of: a TNF signaling pathway inhibitor (lenalidomide: 1 μM, Cat. #S1029, Selleck), an IL-17 signaling pathway inhibitor (Y-320: 10 μM, Cat. # S7516, Selleck), an mTOR signaling pathway activator (MHY1485: 10 μM, Cat. #S7811, Selleck), a MAPK signaling pathway inhibitor (SB203580: 10 μM, Cat. #S1076, Selleck), and a FoxO signaling pathway inhibitor (AS1842856: 1 μM, Cat. #S8222, Selleck). Combination 2 (C2) is based on C1 formulation, with the addition of a Hippo signaling pathway inhibitor (verteporfin: 1 μg/ml, Cat. #S1786, Selleck) and a Wnt signaling pathway inhibitor (LGK-974: 0.1 μM, Cat. #S7143, Selleck). For direct mechanistic validation, parallel single-compound intervention groups were established: LPS-stimulated A549 cells were treated with each of the 7 individual compounds at the same concentration as in the C1/C2 formulations, respectively.

### 2.3 Cell migration assay: wound healing (scratch) assay

To evaluate the migratory capacity of the cells, a wound healing (scratch) assay was performed. Briefly, cells were seeded in 6-well plates at a density of 5 × 10^5^ cells/well and cultured until they reached 90–100% confluency. A sterile 200 µL pipette tip was used to create a straight scratch in the cell monolayer to simulate a tissue wound. The wells were gently washed with phosphate-buffered saline (PBS) to remove detached cells and debris. Fresh serum-free medium was added to minimize the influence of cell proliferation on migration. Images of the scratch were captured at 0, 2, 5, and 7 days using an inverted phase-contrast microscope (10× objective). The wound area was quantified via ImageJ software (National Institutes of Health, USA).

### 2.4 Animal model and animal groups

Male C57BL/6J mice (8–10 weeks old, 22–25 g) were purchased from Beijing Charles River Laboratory Animal Technology Co., Ltd. (License: NO.4225MA4F243U403609). All mice were housed in a specific pathogen-free (SPF) environment for one week of acclimatization, with a controlled temperature of 20~25°C, a relative humidity of 30%-70%, and a 12-h light/dark cycle. Animals were allowed free access to standard laboratory chow and water. All experimental protocols were approved by the Institutional Animal Ethics Committee of Wuhan Servicebio Technology Co., Ltd (Approval No. 2025271) and were reported in accordance with the ARRIVE guidelines (Animals in Research: Reporting *In Vivo* Experiments) (33).

Sepsis mouse model was induced using the cecal ligation and puncture (CLP) procedure. The mice were fasted for 12h pre-surgery, with free access to water, then anesthetized with intraperitoneal injection of tribromoethanol (240 mg/kg, Cat. #T48402, Sigma-Aldrich). A 1.5cm midline abdominal incision was made to exteriorize the cecum. Approximately 75% of the cecum (distal to the ileocecal valve) was ligated with a 4–0 silk suture (Cat. #S5028, Ethicon), then punctured twice with a 21G sterile puncture needle (Cat. #305106, BD). Approximately 5 mg of fecal content was extruded to ensure sepsis induction, before returning the cecum to the peritoneal cavity and closing the abdominal wall in two layers with 4–0 silk suture. Sham-operated mice underwent identical laparotomy, cecum exteriorization, and closure, but without ligation or puncture. All animals received a subcutaneous injection of pre-warmed sterile saline (1 mL) for fluid resuscitation immediately after surgery. Sepsis severity was evaluated at 6h post-surgery, defined by: body temperature maintained between 35~36 C (hypothermia indicative of severe sepsis), ≥90% survival rate (to exclude acute death from surgical complications), and serum IL-6 > 800 pg/mL with TNF-α > 1000 pg/mL (detected by ELISA, consistent with established severe sepsis model standards).

Mice were randomly assigned to eight groups: Sham, CLP, Verteporfin, LGK-974, C1, C1+Verteporfin, C1+LGK-974, C2. For each group, 10 mice were used for survival analysis (biological replicates), and 5 mice were used for organ function detection, histopathological analysis, qRT-PCR, and Western blot (biological replicates), with 3 technical replicates for each detection assay. After dose gradient pre-experiments, single inhibitors (verteporfin: 0.1 mg/kg; LGK-974: 5 mg/kg), C1 combination (lenalidomide: 20 mg/kg, Y-320: 10 mg/kg, MHY1485: 5 mg/kg, SB203580: 10 mg/kg, and AS1842856: 3 mg/kg) or C2 combination (C1, verteporfin: 0.1 mg/kg, and LGK-974: 5 mg/kg) were administered via intraperitoneal injection one hour after sepsis induction. The CLP and Sham groups received the same volume of saline. All mice designated for sample collection were sacrificed 24h after surgery, followed by blood and tissue harvesting.

### 2.5 Assessment of organ function injury

Serum was separated from whole blood via centrifugation and stored at -80 C until analysis. Levels of serum aspartate aminotransferase (AST), alanine aminotransferase (ALT), blood urea nitrogen (BUN), and creatinine (CREA) were quantified using a fully automated biochemical analyzer (model specified) according to the manufacturer’s protocols. The degree of organ function injury in each group was compared based on the detected biomarker levels.

### 2.6 Histopathological analysis

Heart, liver, lung, and kidney tissues were harvested at the designated time points, fixed in 4% paraformaldehyde for 24 hours, then processed through a graded ethanol series, cleared in xylene, and embedded in paraffin. Tissue sections of 4 μm thickness were cut and stained with hematoxylin and eosin (H&E). Histopathological changes were examined under a light microscope at 40× magnification by an investigator blinded to the group assignments.

Histopathological injury was quantitatively assessed using a scoring system: 0 (no injury), 1 (<25%), 2 (25–50%), 3 (51–75%), and 4 (>75%). The sum of all pathological feature scores for each organ constitutes the total injury score. The pathological changes of each tissue are as follows:

Heart: edema, inflammatory cell infiltration (total score: 0–8);

Liver: edema, fatty degeneration, congestion, inflammatory cell infiltration (total score: 0–16);

Lung: congestion, hemorrhage, alveolar wall thickening, inflammatory cell infiltration (total score: 0–16);

Kidney: tubular epithelial edema, tubular dilation, connective tissue hyperplasia, congestion, inflammatory infiltration (total score: 0–20).

### 2.7 Safety assessment of C2 combination

The safety of C2 combination was evaluated in CLP-induced septic mice, with the dosing regimen fully consistent with the therapeutic experiment. Mice were randomly divided into 4 groups (n=5 per group): Sham group (sham operation + normal saline), Vehicle group (CLP model + equivalent drug solvent), C2-Therapeutic group (CLP model + therapeutic dose of C2), and C2-High group (CLP model + 3× supra-therapeutic dose of C2). All agents were administered via intraperitoneal injection once daily for 7 consecutive days. Body weight and general behavioral state were monitored daily at a fixed time point; orbital venous blood was collected daily to separate serum, and serum levels of ALT, AST, BUN, CREA were detected using an automatic biochemical analyzer.

### 2.8 Enzyme-linked immunosorbent assay

To determine cytokine levels in cell experiments, TNF-α (Cat. #88-7346-22, Invitrogen) and IL-6 (Cat. #88-7066-22, Invitrogen) ELISA kits were used in accordance with the manufacturer’s specifications. For animal experiments, to further detect cytokine levels in serum, IL-1β (Cat. #88-7013A-76, Invitrogen), TNF-α (Cat. #88-7324-22, Invitrogen), and IL-6 (Cat. #88-7064-22, Invitrogen) ELISA kits were employed following the manufacturer’s instructions. To validate the modulation of multi-omics identified dysregulated inflammatory signaling pathways (TNF, IL-17, MAPK, mTOR, FoxO, Hippo, Wnt), we detected the phosphorylation levels of key pathway molecules using commercial mouse-specific PathScan^®^ ELISA kits (Cell Signaling Technology, CST): phospho-NF-κB p65 (Ser536, Cat. #7173), phospho-p38 MAPK (Thr180/Tyr182, Cat. #7946), phospho-STAT3 (Tyr705, Cat. #7300), phospho-mTOR (Ser2448, Cat. #7976), phospho-FoxO3a (Ser253, Cat. #20922). Briefly, 96-well plates pre-coated with capture antibody were used. All samples and provided standards were assayed in triplicate. After blocking, samples and serially diluted standards were added to the wells and incubated. Following a washing step to remove unbound substances, a biotin-conjugated detection antibody was added, followed by incubation with streptavidin-horseradish peroxidase (HRP). Finally, the reaction was developed using a tetramethylbenzidine (TMB) substrate solution. The enzymatic reaction was stopped by adding stop solution, and the optical density (OD) of each well was immediately measured at 450 nm with a reference wavelength of 570 nm using a microplate reader (Cat. #680, Bio-Rad). The relative phosphorylation level of each target protein was calculated by normalizing the OD value of each sample to the mean OD value of the Sham group. Statistical analysis was performed using one-way ANOVA with *post hoc* Tukey’s test for multi-group comparisons.

### 2.9 Quantitative real-time PCR

Total RNA was extracted from the collected samples according to the instructions for TRIzol (Cat. #R401-01, Vazyme). For mRNA quantification, cDNA was subsequently synthesized from the extracted RNA using the Reverse Transcription Kit HiScript III RT SuperMix (Cat. #R323-01, Vazyme), with *Gapdh* as the internal control. For miRNA quantification, cDNA was synthesized using the stem-loop method with the Mir-X miRNA First - Strand Synthesis Kit (Cat. #638313, Takara), with U6 snRNA as the internal control. The mRNA expression levels of pro-inflammatory factors (*Il1b*, *Il6*, *Nos2*, *Tnfa*), anti-inflammatory factors (*Il10*, *Arg1*, *Tgfb*, *Nos3*), core genes of dysregulated signaling pathways (*Rela*, *Mapk14*, *Stat3*, *Mtor*, *Foxo3a*, *Yap1*, *Wwtr1*, *Ctnnb1*), cell type-specific marker genes of immune cell populations (*Ly6g*, *S100a8*, *Csf3r*, *Ly6c*, *Cd14*, *Ccr2*, *Cd19*, *Ms4a1*, *Cd79a*, *Ncr1*, *Klrd1*, *Gzmb*), and miRNA target genes (*Rictor*, *Akt2*, *Frs2*, *Mtor*, *Fgfr3*, *Ppp3ca*, *Pik3r1*, *Fgf18*, *Btg2*, *Map3k7*, *Mapk1*, *Fosl2*, *Rela*, *Mapk9*, *Irak1*, *Il6*, *Mapk8*, *Hdac2*) were quantified using the Real-Time Fluorescence Quantitative PCR Kit ChamQ SYBR qPCR Master Mix (Cat. #Q711-02, Vazyme), following the manufacturer’s protocol. The primer sequences are provided in **Supplementary Table S1**, **S2**. Relative gene expression was calculated using the 2^-^ΔΔCt method, and statistical significance was determined by one-way ANOVA with *post hoc* Tukey’s test for multi-group comparisons.

### 2.10 Western blot analysis

At the time of detection, cell and tissue samples were digested with trypsin (Cat. # 25200072, Gibco), then washed 3 times via repeated centrifugation with PBS. After discarding the PBS supernatant, samples were weighed. RIPA cell lysis buffer (Cat. #P0013B, Beyotime) containing 10% protease inhibitor (PMSF, Cat. #ST506, Beyotime) was added to the samples. The mixture was lysed for 20min on ice, then centrifuged at 12000 rpm at 4°C for 10min. Protein concentration was determined using a BCA protein assay kit (Cat. #P0010, Beyotime) following the manufacturer’s instructions. Western blot was performed to analyze the protein expression of key inflammatory mediators, including eNOS, iNOS, and IL-10. Primary antibodies used were: eNOS (Cat. #9586S, CST, 1:1000), iNOS (Cat. #13120S, CST, 1:1000), IL-10 (Cat. #12163S, CST, 1:1000), GAPDH (Cat. #5174S, CST, 1:5000), α-Tubulin (Cat. #2144S, CST, 1:5000). HRP-conjugated goat anti-rabbit IgG (Cat. #7074S, CST, 1:3000) was used as the secondary antibody. GAPDH and α-Tubulin were used as loading controls.

Based on the H&E staining results of histopathology, the liver, lung and kidney tissues with severe structural damage and inflammatory infiltration in the mice were selected. To validate the modulation of multi-omics identified dysregulated Hippo/Wnt regeneration-associated pathways, the protein expression of key molecules of the Hippo/Wnt pathway was detected by Western blot, using the same protein extraction, quantification, and electrophoresis procedures described above. Primary antibodies used for Hippo/Wnt pathway detection were: YAP (Cat. #14074S, CST, 1:1000), TAZ (Cat. #4883S, CST, 1:1000), β-Catenin (Cat. #8480S, CST, 1:1000). α-Tubulin was used as the loading control. The original, uncropped full membrane scans of all the Western blot experiments are provided in **Supplementary Figure S1**–**S8**.

### 2.11 Statistical analysis

Statistical analyses were performed using GraphPad Prism 9.5.1 software. Survival data were visualized with Kaplan-Meier curves, and intergroup differences were compared using the Log-rank (Mantel-Cox) test. For multi-group comparisons, statistical analyses were performed via one-way ANOVA with *post hoc* Tukey’s test to evaluate statistical differences among the different treatment groups. All quantitative data are expressed as mean ± standard deviation (SD). Significant differences (*p* < 0.05, *p* < 0.01, *p* < 0.001, and *p* < 0.0001) are marked with “*”, “**”, “***”, and “****”, respectively; “ns” indicates no significant difference.

## 3 Results

### 3.1 Single-cell sequencing reveals immune cell composition remodeling and sepsis-specific gene signatures

**Figure 1:**
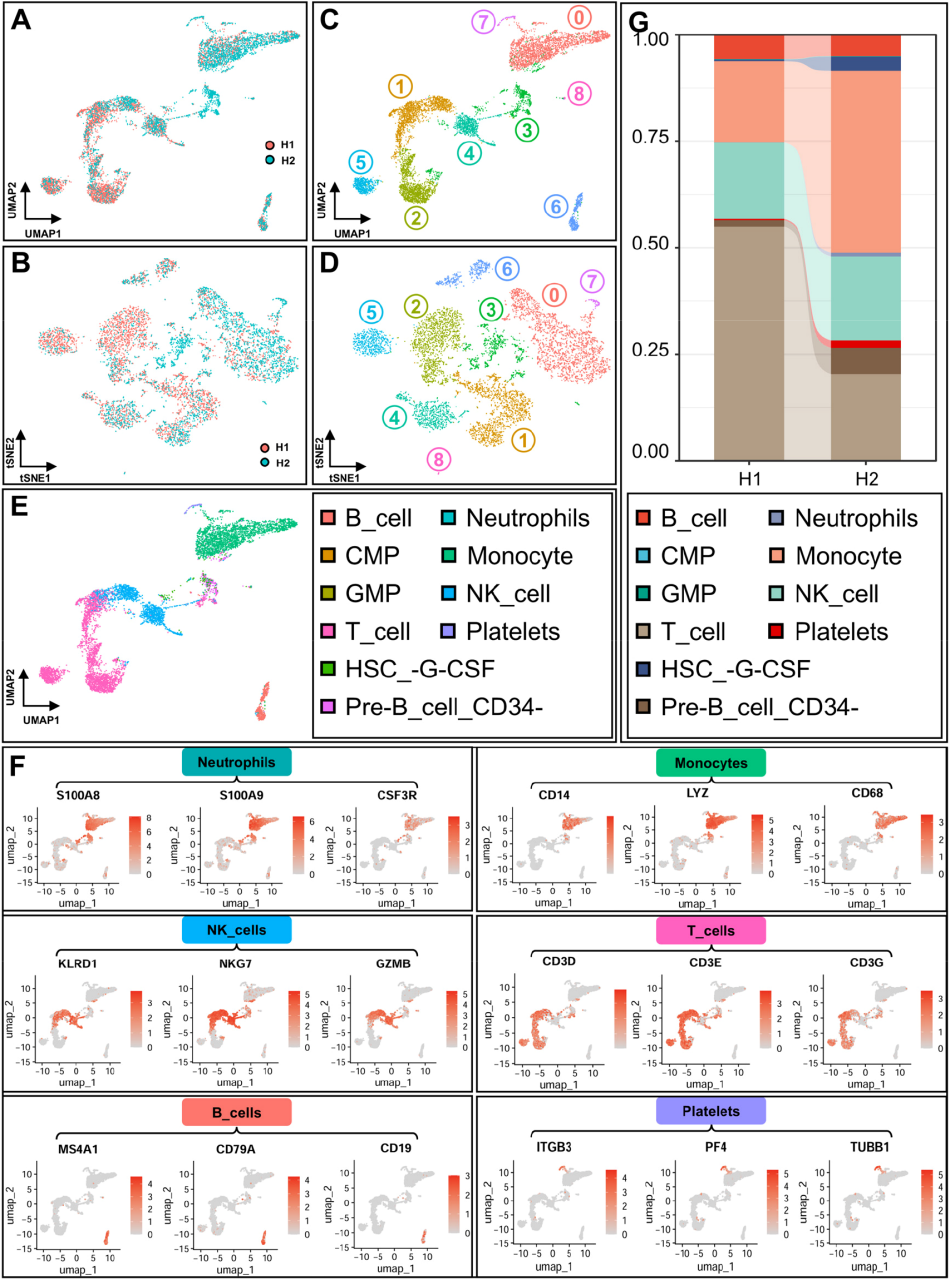
ScRNA-seq reveals immune cell landscape remodeling and sepsis-specific molecular signatures in sepsis. **(A, B)** UMAP plot **(A)** and tSNE plot **(B)** showing sample clustering for the healthy group (H1 in red) and sepsis group (H2 in cyan). **(C, D)** UMAP **(C)** and tSNE **(D)** visualization of all cells clustered into 9 distinct clusters (resolution = 0.1). **(E)** UMAP plot with cell type annotations (determined by SingleR), identifying major lineages including B cells, neutrophils, common myeloid progenitors (CMPs), pre-B cells (CD34−), granulocyte–monocyte progenitors (GMPs), natural killer (NK) cells, G-CSF-treated hematopoietic stem cells (HSCs), platelets, monocytes, and T cells. **(F)** Feature plots on UMAP showing the expression distribution of canonical marker genes for specific cell types: S100A8/S100A9/CSF3R (Neutrophils); CD14/LYZ/CD68 (Monocytes); KLRD1/NKG7/GZMB (NK cells); CD3D/CD3E/CD3G (T cells); MS4A1/CD79A/CD19 (B cells); ITGB3/PF4/TUBB1 (Platelets). **(G)** Stacked bar chart depicting the proportional composition of major cell populations in the H1 and H2 groups, highlighting inter-group differences in cellular distribution.

To delineate the immune landscape of sepsis, scRNA-seq was performed on whole blood samples from 5 healthy individuals (H1) and 5 septic patients (H2) using the GSE224095 dataset. Unsupervised dimensionality reduction via UMAP and tSNE revealed distinct clustering patterns between H1 and H2, with clear separation between groups and consistent aggregation within groups (**Figures 1A, B**). This finding indicated substantial transcriptional heterogeneity between healthy controls and septic patients. Cluster analysis identified nine cell clusters (resolution = 0.1) (**Figures 1C, D**), which were first annotated via the SingleR algorithm, followed by manual validation based on the expression distribution of well-recognized canonical cell-type-specific marker genes (**Figures 1E, F**). The expression patterns of these canonical markers were highly consistent with the annotated cell clusters, indicating strong lineage specificity and clear spatial separation between different immune cell populations on UMAP. Finally, all cells were annotated into 10 major immune cell types, including B cells, neutrophils, common myeloid progenitors (CMPs), pre-B cells (CD34−), granulocyte–monocyte progenitors (GMPs), NK cells, G-CSF-treated hematopoietic stem cells (HSCs), platelets, monocytes, and T cells (**Figure 1E**). Quantitative analysis of cell proportions revealed striking compositional disparities: neutrophils and monocytes were significantly enriched in H2 group, indicating enhanced myeloid cell activity, whereas B cells and NK cells were markedly depleted, suggesting impaired adaptive immunity (**Figure 1G**).

### 3.2 miRNA-seq profiling identifies sepsis-associated regulatory networks and target mRNAs

**Figure 2:**
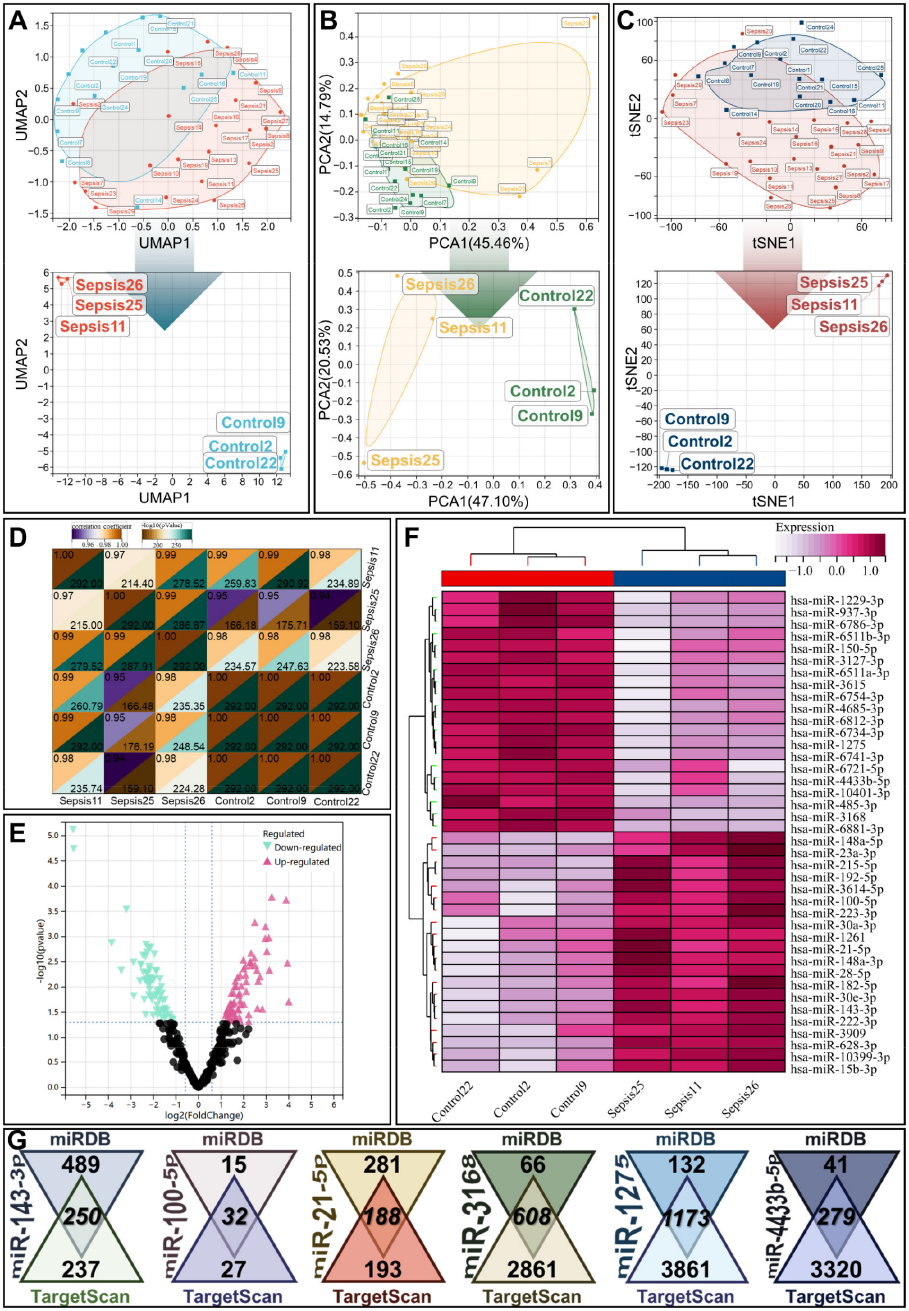
miRNA-seq analysis of whole blood from the healthy and sepsis groups. **(A, C)** UMAP **(A)**, PCA **(B)** and tSNE **(C)** plots showing sample distribution of the healthy control group and sepsis group, with three representative samples selected from each group for visualization (Control: 2, 9, 22; Sepsis: 11, 25, 26). **(D)** Pearson correlation heatmap of samples between the healthy and sepsis groups. **(E)** Volcano plot of differentially expressed miRNAs identified via LIMMA analysis. Thresholds: adjusted *p*-value < 0.05, |log2(fold change)| ≥ 1.5; pink = upregulated miRNAs, cyan = downregulated miRNAs in sepsis. **(F)** Hierarchically clustered heatmap of differentially expressed miRNAs. **(g)** Venn diagram of consensus target mRNAs predicted by the miRDB and TargetScan databases for 6 key dysregulated miRNAs.

miRNA-seq analysis was performed on whole blood samples from 22 septic patients and 15 healthy subjects using the GSE243218 dataset. Dimensionality reduction via UMAP, PCA, and tSNE confirmed good consistency within sample groups, with three representative samples selected from each group for subsequent analysis (Control: 2, 9, 22; Sepsis: 11, 25, 26) (**Figures 2A–C**). Correlation heatmaps demonstrated stronger intragroup correlations in control samples and greater heterogeneity in septic samples, consistent with the complex pathological features of sepsis (**Figure 2D**).

LIMMA-based differential expression analysis revealed significantly dysregulated miRNAs, including hsa-miR-143-3p, hsa-miR-100-5p, hsa-miR-21-5p, hsa-miR-4433b-5p, hsa-miR-1275, and hsa-miR-3168 (**Figures 2E, F**). We performed high-confidence target mRNA prediction for these dysregulated miRNAs using the miRDB and TargetScan databases, and retained only overlapping target genes for further analysis (**Figure 2G**). These miRNAs are known to regulate immune cell activation, inflammatory responses, and apoptosis, highlighting their potential roles in sepsis pathogenesis.

### 3.3 RNA-seq reveals key differentially expressed mRNAs in systemic and tissue-specific inflammatory responses

**Figure 3:**
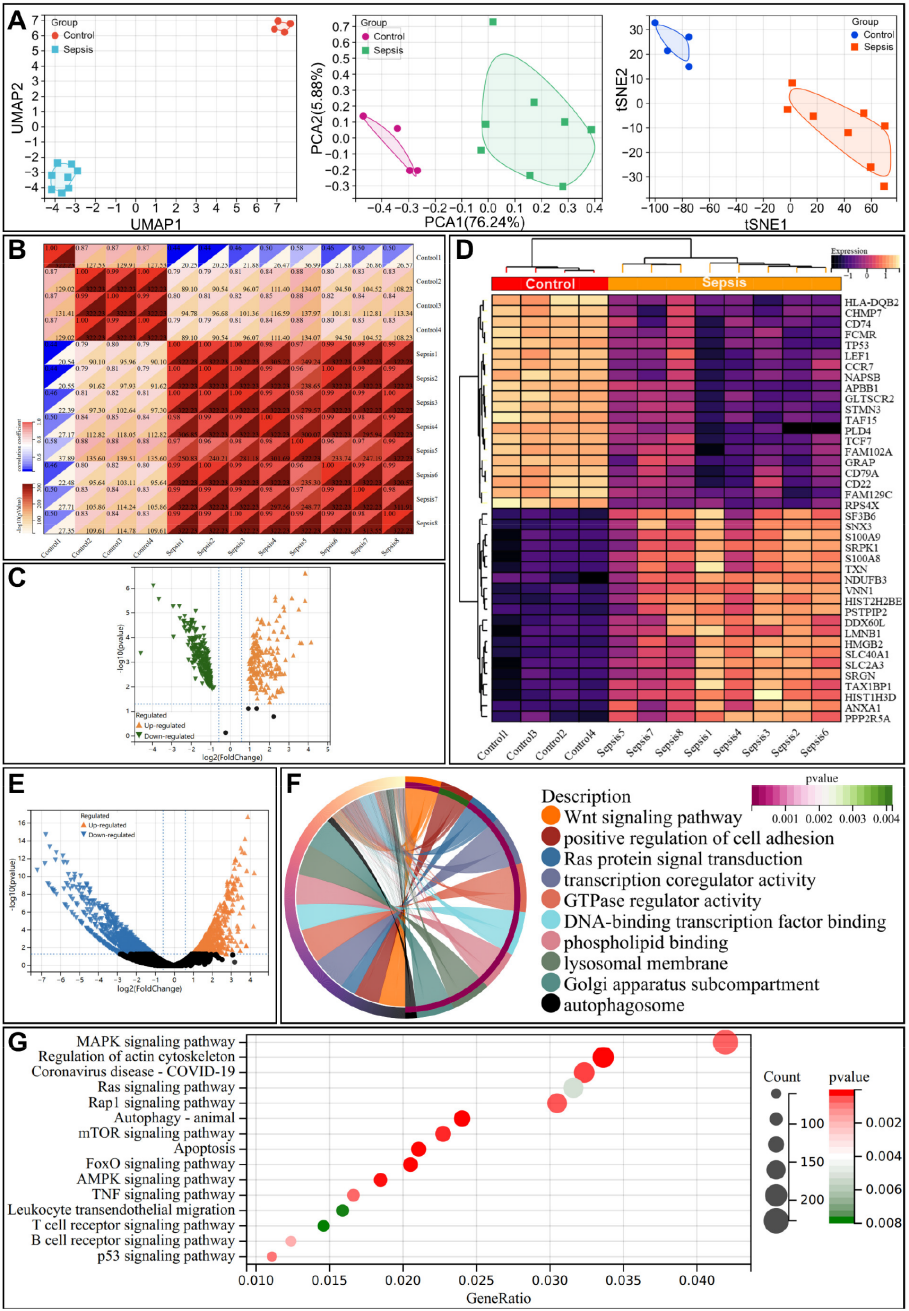
RNA-seq analysis of whole blood and lung tissues from the healthy and sepsis groups. **(A)** Multimodal dimensionality reduction plots (UMAP, PCA, and tSNE) of whole-blood RNA-seq data, showing distinct clustering between healthy and septic groups. **(B)** Pearson correlation heatmap of whole blood samples from the healthy and sepsis groups. **(C)** Volcano plot of differentially expressed mRNAs identified by LIMMA analysis in whole blood samples. Thresholds: adjusted p-value < 0.05, |log2(fold change)| ≥ 1.5; orange = upregulated mRNAs, green = downregulated mRNAs in sepsis. **(D)** Heatmap of differentially expressed mRNAs in whole blood samples. **(E)**: Volcano plot of differentially expressed mRNAs identified by LIMMA analysis in lung tissues (same thresholds as panel **(C, F, G)** Bubble plots of GO functional annotation **(F)** and KEGG pathway enrichment analysis **(G)** of differentially expressed mRNAs in lung tissues. **(F, G)** Bubble plots of GO functional annotation **(F)** and KEGG pathway enrichment analysis **(G)** of differentially expressed mRNAs in lung tissues. Dot size represents the number of enriched genes; color intensity represents the adjusted *p*-value of enrichment.

To validate miRNA target genes and identify tissue-specific changes, RNA-seq analysis was performed on whole blood (GSE232753) and lung tissue (GSE237861) samples. Dimensionality reduction via UMAP, PCA, and tSNE confirmed distinct clustering between healthy and septic groups (**Figure 3A**), and sample correlation analysis validated data quality and reliability of the sequencing data (**Figure 3B**). LIMMA-based differential expression analysis identified 191 upregulated and 228 downregulated mRNAs in whole blood, with the expression of *S100A8*, *S100A9*, and *CD38* significant upregulated (*p* < 0.05) (**Figures 3C, D**).

Differential analysis of lung tissues, a major target of sepsis-induced injury, revealed significant upregulation of *IL6R*, *TNFR*, and *IFNG* (*p* < 0.05) (**Figure 3E**). GO and KEGG enrichment analyses showed that differentially expressed mRNAs were enriched in biological processes related to immune response, inflammation, and cellular signaling pathways, including the Wnt, MAPK, and mTOR pathways (**Figures 3F, G**). These findings underscore the critical involvement of these pathways in sepsis progression.

### 3.4 Multi-omics integration elucidates core molecular pathways in sepsis

**Figure 4:**
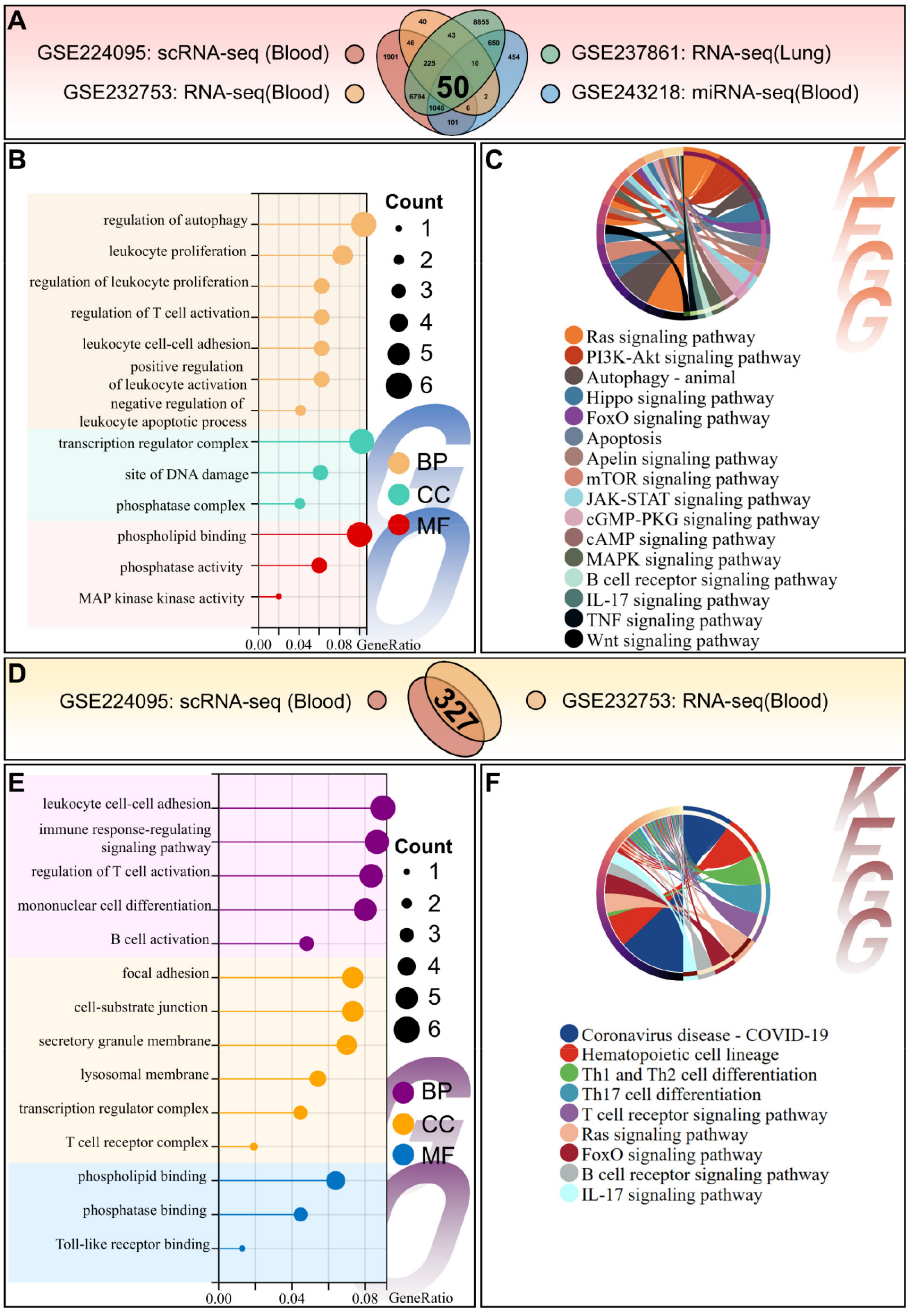
Multi-omics integration analysis of sepsis-associated dysregulated genes and pathways. **(A)** Venn diagram depicting the number of overlapping differentially expressed mRNAs across four datasets: whole-blood scRNA-seq (GSE224095), whole-blood miRNA-seq target mRNAs (GSE243218), whole-blood bulk RNA-seq (GSE232753), and lung tissue RNA-seq (GSE237861). **(B, C)**. GO functional annotation **(B)** and KEGG pathway enrichment analysis **(C)** of the 50 consensus mRNAs shared by all four omics datasets. **(D)** Venn diagram identifying overlapping mRNAs between whole-blood scRNA-seq and whole-blood bulk miRNA-seq datasets. **(E, F)**. GO functional annotation **(E)** and KEGG pathway enrichment analysis **(F)** of consensus mRNAs from scRNA-seq and bulk miRNA-seq. Bar length represents the number of enriched genes; dot size represents the number of enriched genes; color intensity represents the adjusted *p*-value of enrichment.

To identify consensus dysregulated molecules and pathways in sepsis, multi-omics data (scRNA-seq, miRNA-seq, blood RNA-seq, lung RNA-seq) were integrated. Venn diagram analysis revealed 50 common differentially expressed genes across all four datasets (**Figure 4A**). GO enrichment analysis showed these genes were associated with leukocyte proliferation (BP), T cell activation regulation (BP), lysosomal membrane composition (CC), and phospholipid binding (MF) (**Figure 4B**). KEGG pathway analysis indicated enrichment of the FoxO signaling pathway, IL-17 signaling pathway, and B/T cell receptor signaling pathway (**Figure 4C**). These results highlight the synergy between immune hyperactivation and lysosomal dysfunction in sepsis.

**Figure 5:**
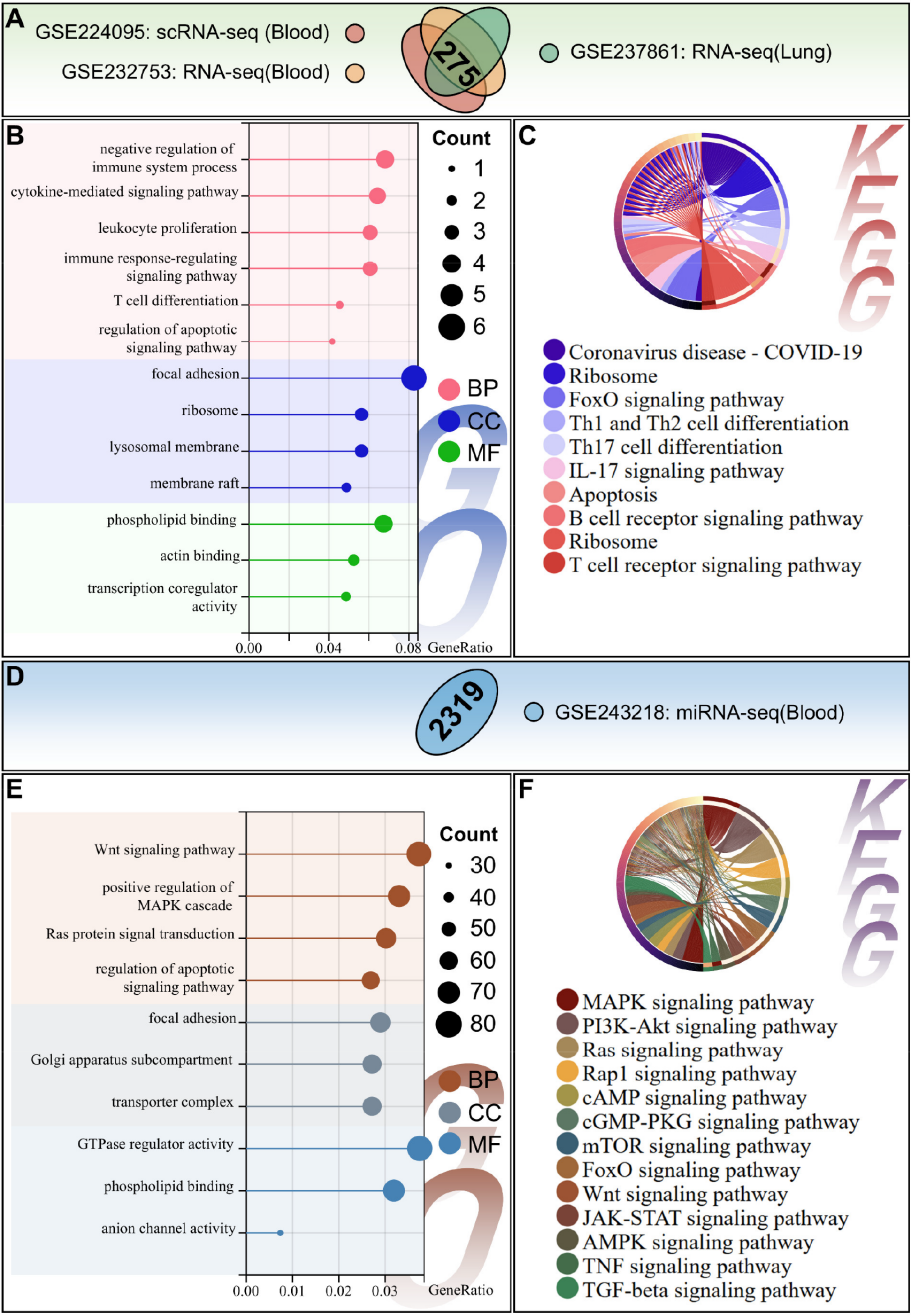
Cross-tissue multi-omics integration and miRNA target gene enrichment analysis. **(A)** Venn diagram of overlapping mRNAs among whole-blood scRNA-seq, whole-blood bulk RNA-seq, and lung tissue RNA-seq datasets. **(B, C)** GO functional annotation **(B)** and KEGG pathway enrichment analysis **(C)** of consensus mRNAs shared by three omics datasets. **(D)** Venn diagram of high-confidence target mRNAs of differentially expressed miRNAs from whole-blood miRNA-seq. **(E, F)** GO functional annotation **(E)** and KEGG pathway enrichment analysis **(F)** of miRNA-targeted mRNAs. Dot size represents the number of enriched genes; color intensity represents the adjusted p-value of enrichment.

Integration of blood-derived omics data (scRNA-seq and RNA-seq) revealed genes enriched in leukocyte cell-cell adhesion (BP) and Toll-like receptor binding (MF) (**Figures 4D, E**). KEGG pathways of these overlapping genes focusing on hematopoietic cell lineage, Th1/Th2/Th17 cell differentiation, T cell receptor signaling, and Ras signaling (**Figure 4F**). Cross-tissue integration of blood and lung RNA-seq data confirmed shared enrichment in immune regulation, inflammation, apoptosis, and signaling pathways (**Figures 5A–C**), providing insights into systemic pathological mechanisms of sepsis. Enrichment analysis of miRNA-seq target genes further validated key dysregulated pathways including the Wnt, MAPK, and TNF signaling pathways (**Figures 5D–F**).

### 3.5 Pathway-targeted small-molecule combinations mitigate sepsis-like phenotypes *in vitro*

**Figure 6:**
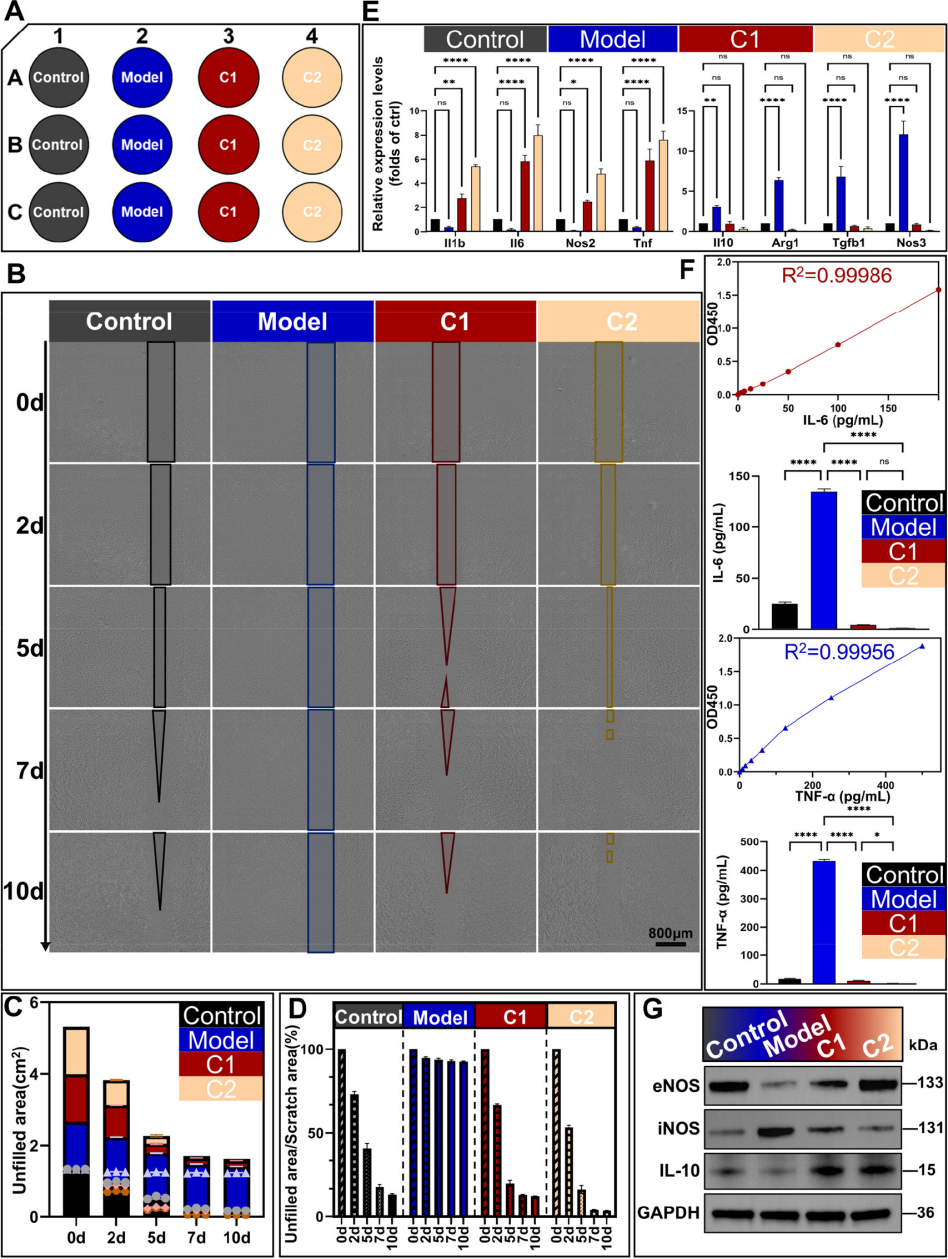
*In vitro* validation of the sepsis cell model and therapeutic effects. **(A)** Experimental design: Control group (untreated A549 cells), Model group (LPS-induced A549 cells), and two small-molecule combination therapies: C1 group (Model + C1 combination) and C2 group (Model + C2 combination). **(B)** Representative images of the scratch wound healing assay at 0 d, 2 d, 5 d, 7 d, and 10 d in each group (scale bar=800 μm). **(C)** Quantification of the unhealed wound area at each time point. **(D)** Wound healing rate (%) = unhealed wound area/initial scratch wound area × 100%. **(E)** qRT-PCR analysis of pro-inflammatory (*Il1b*, *Il6*, *Nos2*, *Tnfa*) and anti-inflammatory/repair-related (*Il10*, *Arg1*, *Tgfb*, *Nos3*) cytokines mRNA levels (*Gapdh* as internal control). **(F)** ELISA detection of IL-6 and TNF-α protein levels in cell supernatant (pg/mL). **(G)** Western blot analyses of eNOS (~133 kDa), iNOS (~131 kDa), IL-10 (~15 kDa), and GADPH (~36 kDa) protein expression in each group. Cropped images were processed uniformly without altering the original data integrity. Statistical analysis: One-way ANOVA with Tukey’s *post-hoc* test **(E, F)**; n = 3 per group; Data are presented as the mean ± SD; *p < 0.05, **p < 0.01, ****p < 0.0001, ns, no significant difference.

Based on the core dysregulated pathways identified via multi-omics integration, two small-molecule combinations were designed: C1 (targeting the TNF, IL-17, mTOR, MAPK, and FoxO pathways) and C2 (C1, Hippo pathway inhibitor verteporfin and Wnt pathway inhibitor LGK-974). An *in vitro* sepsis model was established by stimulating A549 cells with LPS (**Figure 6A**).

Wound healing assays revealed that the unhealed area was 92.5% in the LPS-induced model group on day 10. In comparison, the unhealed area was reduced to 12.4% in the C1-treated group (86.6% reduction) and 3.5% in the C2-treated group (96.2% reduction) (**Figures 6B–D**). C2 exhibited superior healing efficiency, with a 71.8% lower residual wound area than C1 group. qRT-PCR analysis demonstrated that both combinations suppressed pro-inflammatory mediators (*Il1b*, *Il6*, *Nos2*, *Tnfa*) and upregulated anti-inflammatory/repair-related factors (*Il10*, *Arg1*, *Tgfb*, *Nos3*) (**Figure 6E**). Compared with C1, C2 achieved more potent suppression of pro-inflammatory cytokines (90.1%, 99.7%, 98.9% reduction for *Il1b*, *Il6*, *Tnfa*, respectively) and induction of anti-inflammatory mediators (15.8-fold for *Il10*, 58.0-fold for *Tgfb*) than did C1. ELISA and Western blot analyses further confirmed these trends: C2 treatment resulted in greater reductions in IL-6 and TNF-α levels, and consistent regulation of iNOS and IL-10 protein expression (**Figures 6F, G**).

To validate the direct modulatory effect of each compound on its target pathway and establish a causal link between the therapeutic agents and the identified dysregulated pathways, we performed single-compound intervention experiments in the LPS-induced A549 cell model (**Supplementary Figure S9**). qRT-PCR analysis confirmed that each compound significantly regulated the core gene of its corresponding target pathway (all *p* < 0.0001). C1 combination showed significantly stronger regulatory effects on all inflammatory pathway genes than corresponding single-compound treatments (*p* < 0.05), demonstrating the synergistic benefit of simultaneously blocking the inflammatory cascade via multiple targets. Compared with the corresponding single-compound treatments, C2 combination exerted significantly greater regulatory effects on all genes (*p* < 0.05), reflecting the synergistic effect of co-targeting inflammatory and regenerative pathways. These results establish a direct causal relationship between each compound and its target pathway.

### 3.6 Small-molecule combinations alleviate sepsis-induced multi-organ dysfunction, reprogram organ-specific inflammatory responses, and improve survival *in vivo*

**Figure 7:**
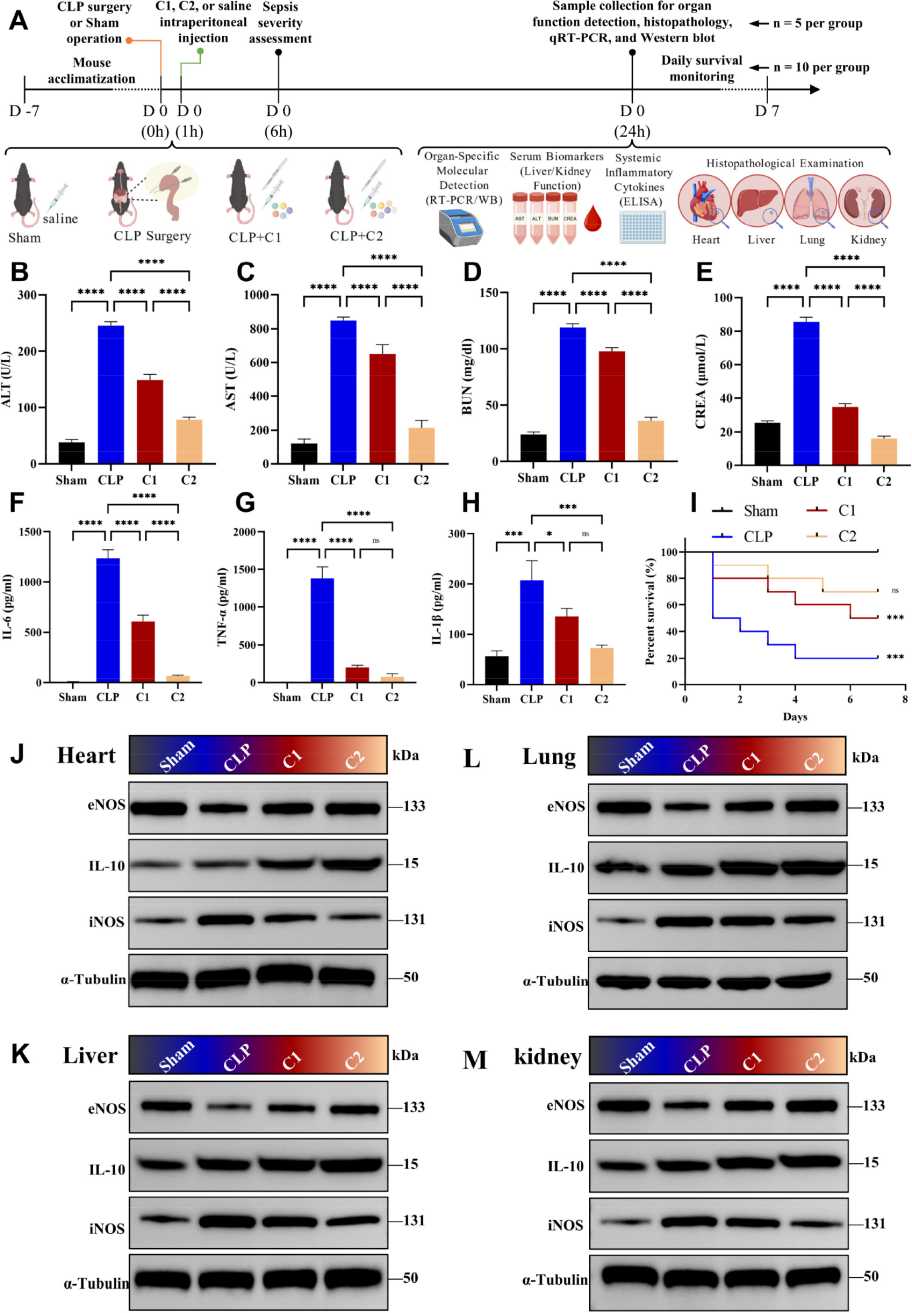
*In vivo* effects of C1/C2 combinations on liver and kidney functions, organ-specific inflammation, and survival. **(A)** Experimental timeline for the *in vivo* CLP mouse sepsis model. **(B–E)**. Serum levels of hepatic and renal function biomarkers in mice from each group: **(B)** AST, **(C)** ALT, **(D)** BUN and **(E)** CREA. **(F, H)** ELISA detection of systemic pro-inflammatory cytokines in mouse serum: **(F)** IL-6, **(G)** TNF-α, **(H)** IL-1β. **(I)**: Kaplan-Meier survival curves for 7 days post-CLP surgery with Log-rank (Mantel-Cox) test (n =10 per group). **(J–M)**. Western blot analyses of eNOS (~133 kDa), IL-10 (~15 kDa), iNOS (~131 kDa), and α-Tubulin (~50 kDa, loading control) protein expression in heart **(J)**, liver **(K)**, lung **(L)**, and kidney **(M)** tissues. Cropped images were processed uniformly without altering the original data integrity. Statistical analysis: One-way ANOVA with Tukey’s *post-hoc* test **(B–H)**; n = 5 per group; Log-rank (Mantel-Cox) test **(I)**; Data are presented as the mean ± SD; *p < 0.05, ***p < 0.001, ****p < 0.0001, ns, no significant difference.

A murine sepsis model was established via cecal ligation and puncture (CLP), with mice randomized into four groups: Sham, CLP, C1, and C2 (**Figure 7A**). Serum biomarker analysis showed that CLP-induced sepsis significantly increased the levels of AST, ALT, BUN, and CREA, which are classic biomarkers of hepatic and renal dysfunction. C2 treatment significantly reduced the levels of these biomarkers (*p* < 0.05), with a significantly better protective effect than C1 (*p* < 0.05) (**Figures 7B–E**; **Supplementary Table S3**). ELISA analysis confirmed that CLP mice exhibited marked systemic inflammation, with high circulating levels of pro-inflammatory cytokines (IL-6: 1239.59 pg/mL, TNF-α: 1383.42 pg/mL, IL-1β: 207.36 pg/mL). Both C1 and C2 treatment significantly suppressed this systemic inflammatory response (*p* < 0.05). C2 achieved greater reductions (e.g., 94.26% decrease in IL-6) compared to C1 (~51.05% decrease) (**Figures 7F–H**). Western blot analysis confirmed that CLP mice presented elevated iNOS expression and reduced eNOS expression across all four examined organs. In contrast, C2 treatment downregulated the pro-inflammatory driver iNOS, and upregulated the anti-inflammatory/protective mediator eNOS and IL-10 (**Figure 7J–M**). Survival analysis revealed that C2 treatment improved 7-day survival rate of septic mice to 70%, compared with 20% in the untreated CLP group and 50% in the C1 group (**Figure 7I**).

Critically, the therapeutic efficacy of C2 is accompanied by a favorable preclinical safety profile, a core prerequisite for clinical translation. A 7-day continuous administration assay showed that C2 was well-tolerated at both therapeutic and 3× supra-therapeutic doses in CLP-induced septic mice, with no detectable systemic, hepatic, or renal toxicity, and no dose-dependent adverse reactions (**Supplementary Figure S10**).

**Figure 8:**
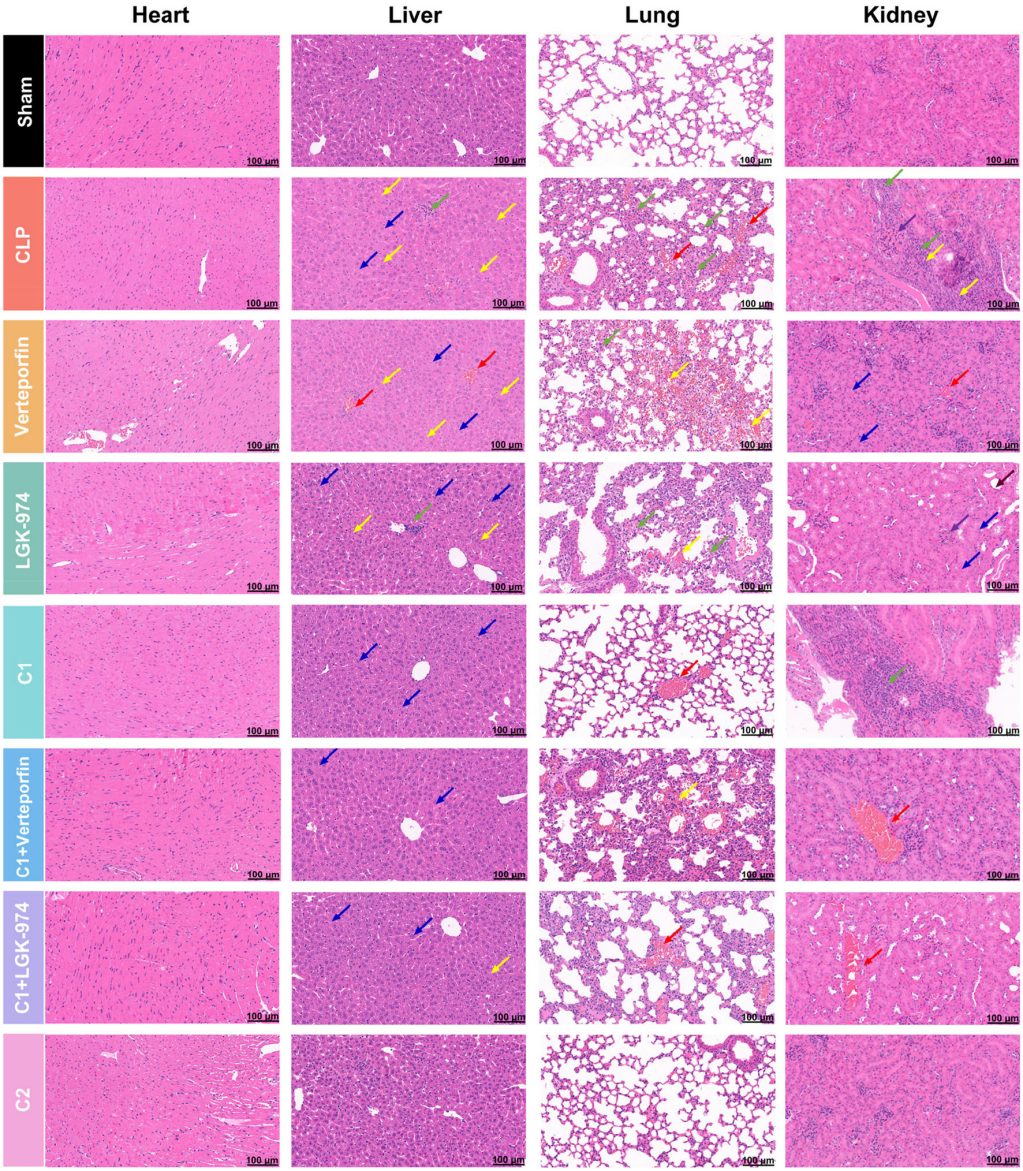
H&E staining of heart, liver, lung, and kidney sections from all eight groups (scale bar = 100 μm). Heart: all structures were clear, and no obvious abnormalities were observed. Liver: edema (blue arrow), fatty degeneration (yellow arrow), congestion (red arrow), inflammatory cell infiltration (green arrow); Lung: hemorrhage (yellow arrow), congestion (red arrow), inflammatory cell infiltration (green arrow); Kidney: glomerular mesangial matrix hyperplasia (purple arrow), renal tubule dilation (brown arrow), tubular epithelial edema (blue arrow), connective tissue hyperplasia (yellow arrow), congestion (red arrow), inflammatory cell infiltration (green arrow).

**Figure 9:**
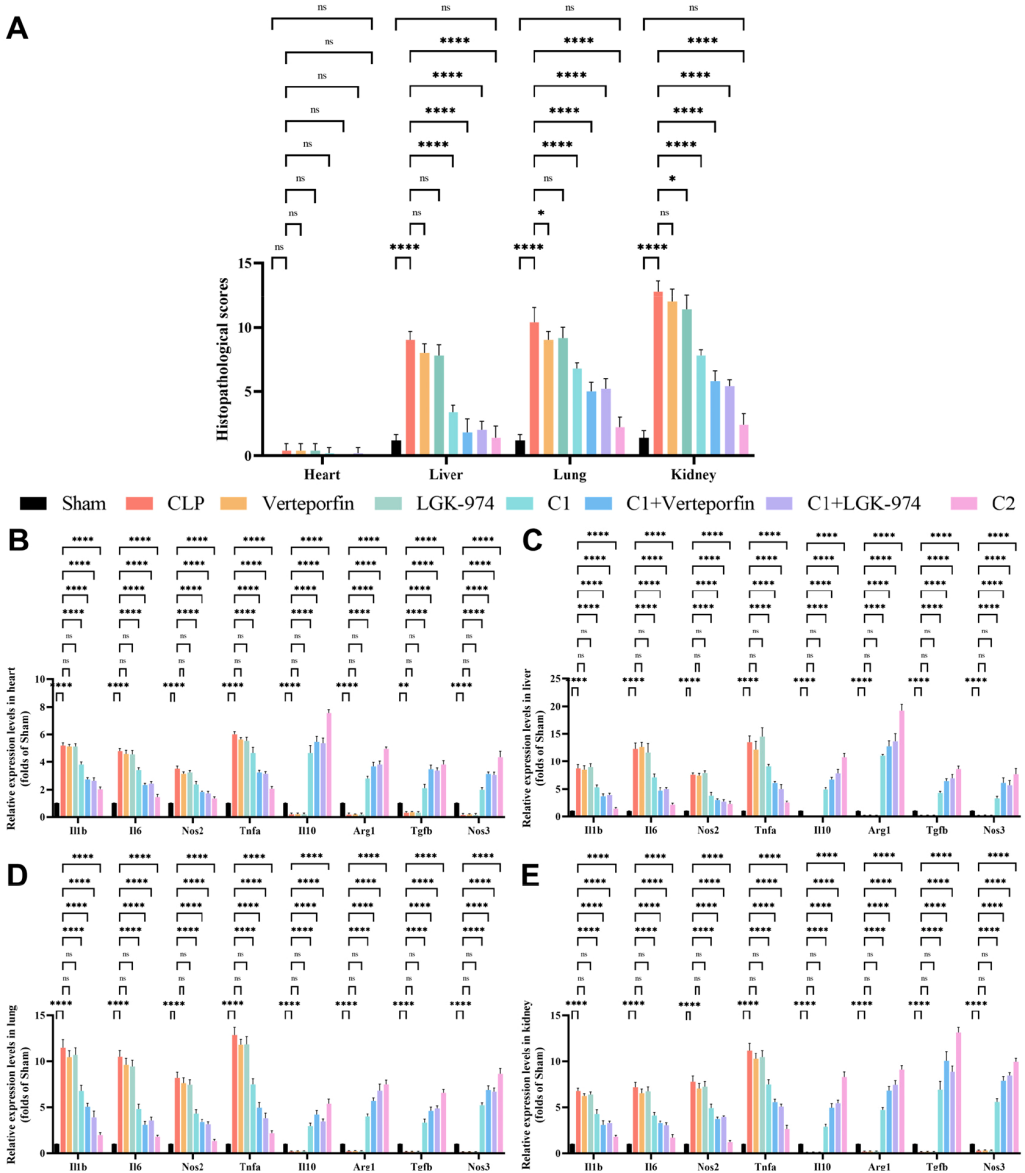
C1/C2 combinations ameliorate organ-specific inflammation response in septic mice. **(A)** Quantitative histopathological injury scores of heart, liver, lung, and kidney tissues from all eight groups, scored in a single-blinded manner according to the standardized scoring system. **(B–E)**. Relative mRNA expression levels of pro-inflammatory genes (*Il1b*, *Il6*, *Nos2*, *Tnfa*) and anti-inflammatory/repair-related genes (*Il10*, *Arg1*, *Tgfb*, *Nos3*) in heart **(A)**, liver **(B)**, lung **(C)**, and kidney **(D)** tissues, with *Gapdh* as the internal reference. Statistical analysis: One-way ANOVA with Tukey’s *post-hoc* test **(A–D)**; n = 5 per group; Data are presented as the mean ± SD; *p < 0.05, **p < 0.01, ****p < 0.0001, ns, no significant difference.

To further investigate whether the enhanced efficacy of C2 is stem from a single added component or the synergistic effect of combinations, we supplemented additional groups treated with verteporfin alone (verteporfin group), LGK-974 alone (LGK-974 group), C1 combined with verteporfin (C1+verteporfin group), and C1 combined with LGK-974 (C1+LGK-974 group) in the *in vivo* CLP sepsis model. Histopathological analysis and quantitative scoring of heart, liver, lung, and kidney tissues were performed across all eight groups (**Figures 8**, **9A**; **Supplementary Tables S4**–**S7**). CLP surgery induced severe structural disruption, inflammatory cell infiltration, edema, and congestion in all examined organs, with significantly elevated pathological injury scores (*p* < 0.0001). Verteporfin or LGK-974 alone treatment did not significantly ameliorate these pathological changes (all *p* > 0.05), whereas C1+verteporfin or C1+LGK-974 group further reduced the pathological scores, with a significantly better effect than C1 alone (*p* < 0.05). C2 treatment exhibited the most potent protective effect, with pathological scores close to the Sham group (all *p* > 0.05) and significantly lower than all other treatment groups (*p* < 0.001).

To investigate the tissue-specific regulatory effects of the combinations and validate the synergistic effect of C2, mRNA expression of key inflammatory and repair-related regulators was quantified in heart, liver, lung, and kidney tissues. CLP surgery significantly upregulated pro-inflammatory genes (*Il1b*, *Il6*, *Nos2*, *Tnfa*) and downregulated anti-inflammatory/repair-related genes (*Il10*, *Arg1*, *Tgfb*, *Nos3*) in all four organs (*p* < 0.0001) (**Figures 9B–E**). Single treatment with verteporfin or LGK-974 alone showed no significant regulatory effect on the expression of these pro-inflammatory or anti-inflammatory/repair-related genes in any examined organs (all *p* > 0.05 p), with only a negligible non-significant trend of mild regulation. These results confirmed that the Hippo/Wnt pathway inhibitors alone have no significant effect on the sepsis-induced inflammatory cascade. Notably, the combination of C1 with verteporfin or C1 with LGK-974 achieved a significantly better regulatory effect than C1 alone (all *p* < 0.05). C2 treatment exerted a more robust regulatory effect: it suppressed pro-inflammatory gene expression levels, and further promoted the induction of anti-inflammatory and repair-related genes (*p* < 0.0001).

### 3.7 C1 and C2 combinations modulate multi-omics identified dysregulated TNF/IL-17/MAPK/FoxO/mTOR/Hippo/Wnt pathways in septic mice

To directly validate the regulatory effect of dual-pathway strategy on the multi-omics identified dysregulated pathways (TNF, IL-17, MAPK, mTOR, FoxO, Hippo, Wnt), we detected the phosphorylation levels of core inflammatory pathway molecules in the liver, lung, and kidney tissues via ELISA, the mRNA expression of all pathway core genes via qRT-PCR, and the protein expression of key Hippo/Wnt pathway molecules via Western blot.

**Figure 10:**
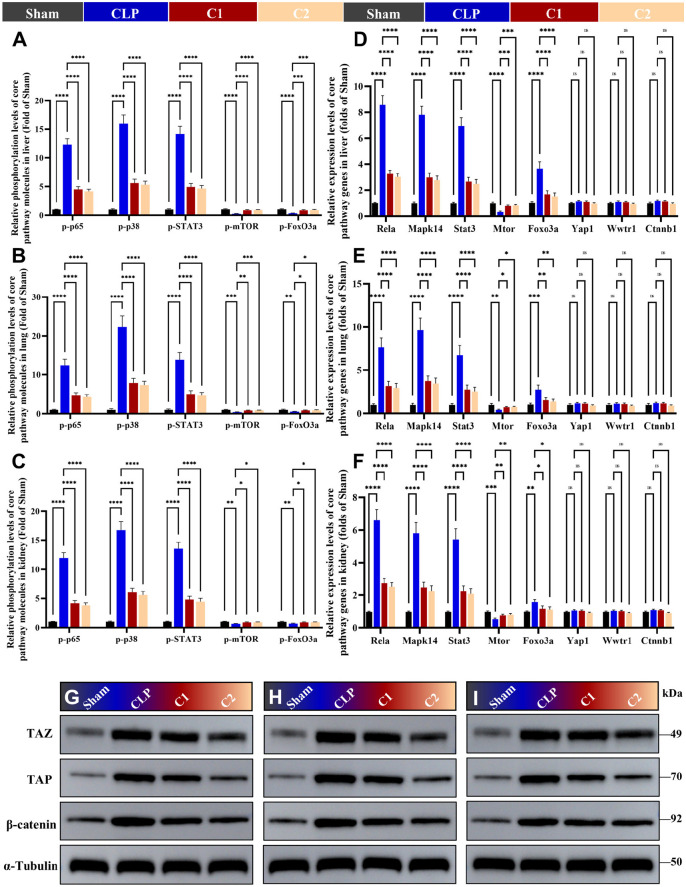
C1 and C2 combinations modulate the dysregulated TNF/IL-17/MAPK/FoxO/mTOR/Hippo/Wnt pathways in septic mice. **(A–C)** ELISA detection of the phosphorylation levels of core pathway molecules (p-p65, p-p38, p-STAT3, p-mTOR, p-FoxO3a, p-YAP, p-TAZ, active β-catenin) in the liver **(A)**, lung **(B)**, and kidney **(C)** tissues. **(D–F)** Relative mRNA expression of core pathway genes (*Rela*, *Mapk14*, *Stat3*, *Mtor*, *Foxo3a*, *Yap1*, *Wwtr1*, *Ctnnb1*) in the liver **(D)**, lung **(E)**, and kidney **(F)** tissues. G-I: Western blot analyses of YAP (~75 kDa), TAZ (~55 kDa), β-catenin (~92 kDa), and α-Tubulin (~50 kDa, loading control) protein expression in the liver **(G)**, lung **(H)**, and kidney **(I)** tissues from Sham, CLP, C1, and C2 groups. Cropped images were processed uniformly without altering the original data integrity. Statistical analysis: One-way ANOVA with Tukey’s *post-hoc* test **(A–F)**; n = 5 per group; Data are presented as the mean ± SD; *p < 0.05, **p < 0.01, ***p < 0.001, ****p < 0.0001, ns, no significant difference.

For the core inflammatory pathways, CLP surgery induced significant dysregulation of these pathways in all three examined organs, as evidenced by the marked increase in phosphorylation levels of p-p65, p-p38, and p-STAT3, and the marked decrease in phosphorylation levels of p-mTOR and p-FoxO3a (all *p* < 0.0001) (**Figures 10A–C**). Notably, FoxO3a is a transcription factor whose function is negatively regulated by phosphorylation: phosphorylation of FoxO3a leads to its nuclear export and proteasomal degradation, thus elevated p-FoxO3a indicates inhibition of FoxO pathway transcriptional activity. Reduced p-FoxO3a in CLP mice reflects excessive activation of the FoxO pathway, which drives sepsis-induced inflammation and organ damage. Both C1 and C2 treatment significantly reversed the over-activation of TNF/IL-17/MAPK/FoxO pathways, and inhibition of mTOR activity (all *p* < 0.0001) (**Figures 10A–C**). qRT-PCR results further confirmed these findings, demonstrating that C1 and C2 significantly reversed the aberrant upregulation of *Rela*, *Mapk14*, *Stat3*, *Foxo3a* expression, and the aberrant downregulation of *Mtor* expression in septic mice (all *p* < 0.05) (**Figures 10D–F**).

The core regulatory mechanism of Hippo/Wnt pathways relies on post-translational phosphorylation modification rather than transcriptional regulation, thus there was no significant difference in the total mRNA levels of *Yap1*, *Wwtr1*, and *Ctnnb1* among all groups (*p* > 0.05), which is consistent with established regulatory patterns of these pathways (**Figures 10D–F**). Western blot results further confirmed these findings, CLP surgery induced significant upregulation of YAP, TAZ, and β-catenin protein expression, indicating aberrant activation of the Hippo and Wnt pathways in sepsis-induced organ damage. Compared with C1, C2 treatment significantly reversed the CLP-induced downregulation of YAP and TAZ, and suppressed the upregulation of YAP, TAZ, and β-catenin in all three examined organs (**Figures 10G–I**).

These results provide direct experimental validation: C1 specifically targets and inhibits the over-activated inflammatory pathways and reverses mTOR/FoxO axis dysregulation in sepsis, while C2 retains the full regulatory effect of C1 and additionally modulates the dysregulated Hippo/Wnt regeneration-associated pathways, confirming the core mechanism of our dual-pathway therapeutic concept.

### 3.8 C2 treatment reverses sepsis-induced immune cell population remodeling and validates miRNA-mRNA regulatory networks in septic mice

**Figure 11:**
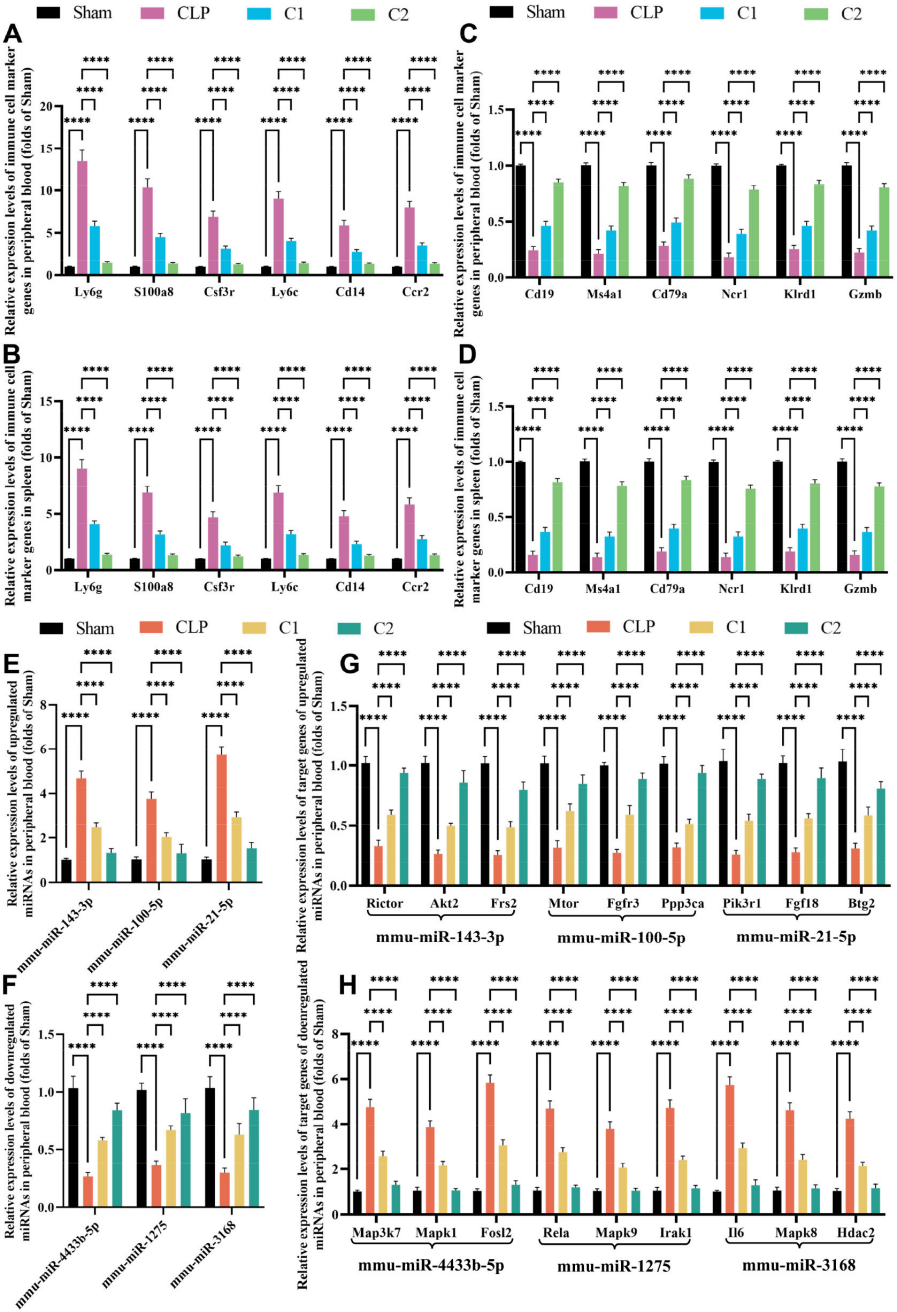
C1 and C2 combinations reverse sepsis-induced immune cell population remodeling in peripheral blood and spleen of septic mice. **(A, B)** Relative mRNA expression levels of neutrophil and inflammatory monocyte marker genes (*Ly6g*, *S100a8*, *Csf3r*, *Ly6c*, *Cd14*, *Ccr2*) in peripheral blood **(A)** and spleen tissues **(B–D)**: Relative mRNA expression levels of B cell and NK cell marker genes (*Cd19*, *Ms4a1*, *Cd79a*, *Ncr1*, *Klrd1*, *Gzmb*) in peripheral blood **(C)** and spleen tissues **(D–F)** Relative expression levels of upregulated miRNAs (mmu-miR-143-3p, mmu-miR-100-5p, mmu-miR-21-5p) **(E)** and downregulated miRNAs (mmu-miR-4433b-5p, mmu-miR-1275, mmu-miR-3168) **(F)** in peripheral blood. **(G, H)** Relative mRNA expression levels of target genes for upregulated miRNAs (*Rictor*, *Akt2*, *Frs2*, *Mtor*, *Fgfr3*, *Ppp3ca*, *Pik3r1*, *Fgf18*, *Btg2*) and downregulated miRNAs (*Map3k7*, *Mapk1*, *Fosl2*, *Rela*, *Mapk9*, *Irak1*, *Il6*, *Mapk8*, *Hdac2*) in peripheral blood. Statistical analysis: One-way ANOVA with Tukey’s *post-hoc* test; n = 5 per group; Data are presented as the mean ± SD; ****p < 0.0001, ns, no significant difference.

To validate the effect of our combination therapies on the immune cell landscape dysregulation identified in septic patients via scRNA-seq, we detected the mRNA expression of cell type-specific marker genes for key immune cell populations in peripheral blood and spleen tissues of mice from each group. Consistent with the clinical scRNA-seq findings, CLP-induced septic mice exhibited significant upregulation of neutrophil markers (*Ly6g, S100a8, Csf3r*) and inflammatory monocyte markers (*Ly6c, Cd14, Ccr2*) (all *p* < 0.0001) (**Figures 11A, B**), indicating marked expansion of pro-inflammatory myeloid cells. Meanwhile, the expression of B cell markers (*Cd19, Ms4a1, Cd79a*) and NK cell markers (*Ncr1, Klrd1, Gzmb*) was significantly downregulated (all *p* < 0.0001) (**Figures 11C, D**), reflecting the depletion of adaptive and innate immune effector cells in sepsis. C1 treatment significantly ameliorated the aberrant expression of these immune cell markers, while C2 treatment exerted a significantly superior regulatory effect: it markedly reduced the expression of myeloid cell markers, and restored the expression of B cell and NK cell markers (all *p* < 0.0001) (**Figures 11A–D**). These results confirm that our combination therapies can reverse sepsis-induced immune cell population remodeling, with C2 showing a more robust effect in restoring systemic immune homeostasis, which is consistent with the superior therapeutic efficacy of C2 observed in our study.

To address the regulatory relationship between dysregulated miRNAs and their target mRNAs identified via miRNA-seq, we further validated the expression of 6 key miRNAs and their target genes (predicted by TargetScan and miRDB) in the peripheral blood of mice. Consistent with the clinical sequencing results, CLP surgery induced significant upregulation of mmu-miR-143-3p, mmu-miR-100-5p, and mmu-miR-21-5p (all *p* < 0.0001), and significant downregulation of mmu-miR-4433b-5p, mmu-miR-1275, and mmu-miR-3168 (all *p* < 0.0001). C2 treatment significantly reversed the aberrant expression of these miRNAs (*p* < 0.001) (**Figures 11E, F**). Notably, the expression of miRNAs showed a strict negative correlation with their corresponding target genes. The target genes of upregulated miRNAs (*Rictor*, *Akt2*, *Frs2*, *Mtor*, *Fgfr3*, *Ppp3ca*, *Pik3r1*, *Fgf18*, *Btg2*) and target genes of downregulated miRNAs (*Map3k7*, *Mapk1*, *Fosl2*, *Rela*, *Mapk9*, *Irak1*, *Il6*, *Mapk8*, *Hdac2*) were significantly restored by C1 and C2 treatment (all *p* < 0.0001) (**Figures 11G, H**). These *in vivo* results directly validate the negative regulatory relationship between the dysregulated miRNAs and their target mRNAs identified via clinical multi-omics analysis, and confirm that C2 can normalize the aberrant miRNA-mRNA regulatory network in sepsis.

## 4 Discussion

### 4.1 Multi-omics deciphers sepsis immune dysregulation: a foundation for targeted therapy

Sepsis remains a lethal clinical challenge due to its complex immune dysregulation—characterized by the paradox of hyperactive innate immunity and compromised adaptive immunity—that conventional anti-inflammatory therapies fail to address (5, 34). The present study leverages integrative multi-omics (scRNA-seq, miRNA-seq, bulk RNA-seq) to dissect this heterogeneity, providing a high-resolution map of sepsis-induced immune remodeling. ScRNA-seq analysis identified a hallmark shift in immune cell composition in septic patients: expansion of myeloid cells (neutrophils, monocytes) and depletion of lymphoid cells (B cells, NK cells) (**Figure 1H**). *In vivo* animal experiments further validated this immune remodeling phenotype, and confirmed that our C2 combination therapy can significantly reverse the expansion of pro-inflammatory myeloid cells and the depletion of B/NK cells, restoring immune homeostasis in septic mice (**Figure 10**). These findings establish a direct link between the clinical immune signatures identified via scRNA-seq and the therapeutic effect of our combination strategy. This compositional remodeling aligns with the “two-hit” hypothesis of sepsis pathogenesis, in which an initial cytokine storm driven by myeloid cells is followed by immunoparalysis due to impaired adaptive immunity (35, 36). Mechanistically, the enrichment of neutrophils likely exacerbates tissue damage through the release of neutrophil extracellular traps (NETs) and proteases (37). Meanwhile, monocyte polarization toward the pro-inflammatory M1 phenotype amplifies systemic inflammation via secretion of IL-1β and TNF-α (38). Conversely, the reduction in B cells may stem from myeloid-biased hematopoiesis and TNF-α-induced apoptosis (39). NK cell depletion compromises pathogen clearance and immune homeostasis, which are key factors linked to sepsis severity and poor prognosis (40).

Notably, miRNA-seq analysis complements these cellular insights by uncovering post-transcriptional regulators that directly modulate the immune cell dynamics and molecular signatures observed in scRNA-seq (**Figures 2E, F**). We identified significantly dysregulated miRNAs in septic samples, including hsa-miR-143-3p, hsa-miR-21-5p, and hsa-miR-3168. The target genes of these miRNAs intersect with pathways governing immune cell activation, apoptosis, and cytokine production (34, 41, 42). For instance, hsa-miR-143-3p, which was upregulated in septic patients, has been linked to T cell immunoparalysis (42). This phenomenon directly correlates with scRNA-seq observations of lymphoid cell depletion and impaired adaptive immunity. Mechanistically, hsa-miR-143-3p targets genes involved in T cell receptor signaling, suppressing T cell activation and proliferation to exacerbate adaptive immune suppression at the cellular level. Similarly, hsa-miR-21-5p, a well-characterized pro-inflammatory miRNA (34). It regulates the expression of pro-inflammatory mediators (e.g., IL-6, TNF-α) by targeting negative regulators of NF-κB, reinforcing the myeloid cell hyperactivation and systemic inflammation detected via scRNA-seq. *In vivo* validation in the CLP mouse model further confirmed the strict negative regulatory relationship between these dysregulated miRNAs and their target genes (**Figures 10E–H**). These findings established a direct post-transcriptional regulatory mechanism linking the miRNA dysregulation to the aberrant activation of core pathological pathways in sepsis.

Collectively, these multi-omics findings not only delineate the cellular and molecular basis of sepsis immune dysregulation, but also establish a hierarchical molecular framework for rational therapeutic target selection: the myeloid expansion/lymphoid depletion signature defines the core immune imbalance to be corrected, while the consistently dysregulated signaling pathways across multi-omics layers provide actionable targets for combinatorial therapy design.

### 4.2 Core pathway identification: guiding rational design of small-molecule combinations

Building directly on our multi-omics integration findings, we systematically prioritized actionable therapeutic pathways through the pre-specified 4-step filtering workflow (detailed in the Methods section). It yielded two non-redundant, functionally complementary pathway categories for combinatorial therapy design: classical inflammatory cascades (TNF/IL-17/MAPK/mTOR/FoxO) and regeneration-associated Hippo/Wnt pathways (**Figures 4C**, **5F**). The TNF, IL-17, MAPK, mTOR, FoxO signaling pathways were consistently enriched across all datasets, confirming their role as a central mediator of systemic inflammation in sepsis. C1 exclusively targets these pan-omics validated inflammatory axes, with the goal of correcting the myeloid cell-driven hyperinflammation and immune imbalance identified via scRNA-seq analysis (**Figure 1**). Meanwhile, Hippo/Wnt dysregulation highlights a critical gap in conventional therapies: sepsis-induced tissue damage is driven not only by inflammation, but also by impaired cellular regeneration (43, 44). This dual pathophysiology (hyperinflammation + defective repair), identified via our multi-omics integration, directly motivated the design of Combination 2 (C2). C2 augments C1’s anti-inflammatory components with a Hippo pathway inhibitor (verteporfin) and a Wnt pathway inhibitor (LGK-974), to simultaneously address both core pathological axes of sepsis identified via multi-omics analysis.

Combination C1, which targets the TNF/IL-17/MAPK/FoxO/mTOR pathways, effectively suppressed pro-inflammatory cytokines but had limited effects on tissue repair and survival (**Figures 6**, **7**). Single-compound intervention experiments further validated the rational design of our combinations: each compound directly modulates its target pathway, the superior efficacy of C1 over single inhibitors arises from synergistic blockade of the inflammatory cascade, and the enhanced effect of C2 stems from simultaneous targeting of both inflammatory and regenerative pathways (**Supplementary Figure S9**). In contrast, C2—augmented with the Hippo inhibitor verteporfin and Wnt inhibitor LGK-974—addressed both pillars of sepsis pathology. Molecular validation experiments further confirmed this mechanism: C1 and C2 showed equivalent regulatory effects on the dysregulated TNF/IL-17/MAPK/FoxO/mTOR pathways, while only C2 significantly reversed the dysregulation of Hippo/Wnt pathways in damaged organs (**Figure 10**) and the aberrant miRNA-mRNA regulatory network (**Figures 11E–H**), providing direct experimental evidence for the targeted regulatory effect of C2 on all multi-omics identified dysregulated pathways. Mechanistically, verteporfin activates YAP/TAZ by inhibiting the Hippo pathway, preserving mitochondrial integrity and reducing renal tubular damage (45), while LGK-974 blocks Wnt signaling to mitigate aberrant inflammatory cascades and restore tissue homeostasis (46). The synergy between these inhibitors and C1’s anti-inflammatory components explained C2’s superior efficacy, underscoring the value of omics-guided, multi-pathway targeting for sepsis therapy.

### 4.3 *In vitro* and *in vivo* therapeutic efficacy of C2 directly validates the multi-omics-derived therapeutic mechanism

The therapeutic efficacy of C2 observed *in vitro* and *in vivo* experiments directly validates the mechanistic hypotheses derived from multi-omics findings, establishing a clear causal link between our multi-omics target identification and the observed therapeutic effects.

*In vitro*, C2 significantly accelerated wound closure in LPS-induced A549 cells (96.2% reduction in unhealed area vs. model group). It also potently regulated cytokine balance, reducing IL-1β, IL-6, and TNF-α by >90% and increasing IL-10 and TGF-β by 15.8- and 58.0-fold, respectively (**Figure 6**). This cytokine rebalancing effect directly reverses the core immune signature identified via scRNA-seq: it suppresses the myeloid cell-driven pro-inflammatory phenotype, while restoring the anti-inflammatory and tissue-reparative transcriptional program that is impaired in septic patients (**Figure 1**).

*In vivo*, C2 treatment reduced the levels of hepatic and renal biomarkers (AST/ALT/BUN/CREA), ameliorated multi-organ pathological damage, and improved 7-day survival to 70% (vs. 20% in untreated CLP mice) (**Figure 7**). Notably, single treatment with verteporfin or LGK-974 alone had no significant therapeutic effect in septic mice, while the combination of C1+verteporfin or C1+LGK-974 achieved a significantly better therapeutic effect than C1 alone (*p* < 0.05), but the effect was still inferior to C2 (**Figures 8**, **9**). These findings directly confirm that the enhanced efficacy of C2 stems from the synergistic effect of dual-pathway (inflammatory cascade + Hippo/Wnt regenerative pathway) co-targeting, rather than the effect of a single added component. This is directly supported by molecular validation results, which showed that C2 simultaneously modulates the dysregulated inflammatory pathways (TNF/IL-17/MAPK/FoxO/mTOR) and regeneration-associated pathways (Hippo/Wnt) in multiple damaged organs (**Figure 10**), accompanied by the restoration of organ structure and function (**Figures 7**, **9**). This finding is consistent with recent reports that YAP/TAZ activation (downstream of Hippo inhibition) maintains mitochondrial integrity in renal tubular epithelial cells to reduce damage (45). Inhibiting canonical Wnt signaling leads to direct activation of YAP/TAZ by the non-classical Wnt pathway. This activation forms a positive feedback loop with other regeneration-related pathways (such as Hippo and Notch pathways), working together to enhance regeneration (47). Unlike single-target drugs (e.g., anti-TNF antibodies) that fail to improve survival (48), C2 modulates interconnected pathways to restore immune balance while promoting tissue repair, a dual benefit rarely achieved by single-target therapies.

The significant downregulation of IL-1β, IL-6, iNOS and TNF-α mRNA in all four organs following C2 treatment indicated that verteporfin and LGK-974 do not merely dampen circulating cytokines (**Figures 9B–E**), but actively rewire local transcriptional circuits. The concurrent induction of IL-10, Arg-1, TGF-β and IL-32 implies polarization of reparative M2 macrophages (49, 50), a transition has previously been shown to be YAP-dependent in hepatic macrophages (51). ScRNA-seq analysis revealed that this polarization process was impaired in patients with sepsis, as the main cells involved exhibited a pro-inflammatory myeloid cell phenotype. Western blot analysis confirmed that C2 simultaneously downregulates iNOS and upregulates eNOS/IL-10 at the protein level, providing translational evidence that these normalized transcripts are functionally translated. Importantly, the magnitude of eNOS induction varied by organ (**Figures 7J–M**), mirroring the differential basal expression of Wnt co-receptors across tissues, suggesting context-specific pathway sensitivity. This organotypic response underscores the value of multi-site molecular profiling when evaluating sepsis therapeutics. It also supports the concept that “one-size-fits-all” immunosuppression is less effective than tailored pathway modulation for sepsis.

### 4.4 Innovation of C2 combination therapy compared with existing sepsis immunomodulatory therapies

Our dual-pathway small-molecule combination strategy addresses key inherent limitations of existing sepsis immunomodulatory therapies, with three distinct advantages over current mainstream interventions. First, in terms of therapeutic target design, our strategy moves beyond the single-target, single-pathway intervention mode of existing therapies (32). Instead, it adopts a dual-pathway synergistic design that simultaneously targets the hyperinflammatory response and organ repair disorder. This design is consistent with the pathological essence of sepsis (a combination of immune dysregulation and multi-organ dysfunction) and avoids the clinical drawbacks of single anti-inflammatory therapies, such as incomplete efficacy and increased infection risk (52). Second, in terms of development rationale, the therapeutic targets of our combination strategy are all identified from integrated multi-omics analysis of clinical sepsis patient samples (blood and lung tissue). This ensures that the targets are highly consistent with the actual pathological characteristics of human sepsis. In contrast, most existing preclinical sepsis immunotherapies are developed based solely on animal models, which contributes to the well-documented translational gap between preclinical efficacy and clinical outcomes. Third, in terms of drug type and clinical translatability, our strategy uses a combination of well-characterized, clinically available small-molecule modulators, and our preclinical assessment further confirmed its favorable safety profile in the septic mouse model, with no detectable systemic or organ-specific toxicity after 7 days of continuous administration. In contrast, most existing sepsis immunotherapies are biological agents (e.g., monoclonal antibodies, recombinant proteins) with high development costs, narrow therapeutic windows, poor tissue penetration, and higher risks of infusion-related adverse events. These limitations restrict their clinical application in critically ill sepsis patients with multi-organ damage (12, 53). Overall, our dual-pathway small-molecule strategy combines anti-inflammatory and organ-protective effects. Its clinical translational potential is further enhanced by its patient-derived targets and clinically available small-molecule components.

### 4.5 Limitations and future directions

In terms of translational potential, all components of C2 are well-characterized small molecules, with lenalidomide, verteporfin, and LGK-974 already clinically approved for other indications, which greatly de-risks subsequent drug development; additionally, the small-molecule formulation is well-suited for the acute sepsis setting, offering rapid onset of action, excellent penetration into injured visceral organs, and low immunogenicity, which addresses key limitations of biological immunotherapies widely used in critical care. However, several core pharmacological limitations remain unresolved in our preclinical work: the intraperitoneal administration route used in mice differs from the intravenous route required for clinical sepsis management, and the intravenous bioavailability, pharmacokinetic/pharmacodynamic profiles in patients with sepsis-associated liver and kidney dysfunction, and potential drug-drug interactions with standard sepsis care have not been systematically characterized. For future clinical applications, our follow-up work will first validate the efficacy and safety of the intravenous formulation of C2 in large animal sepsis models that more closely mimic human pathophysiology. We will also optimize the clinical-adapted administration regimen, evaluate the synergistic effect of C2 with standard sepsis care (antimicrobials, hemodynamic resuscitation), and further validate the multi-omics-identified biomarkers for patient stratification.

## 5 Conclusion

In this study, we systematically dissected the immune dysregulation landscape of human sepsis via integrated multi-omics analysis, and identified two core categories of dysregulated pathways driving sepsis progression: classical inflammatory cascades (TNF/IL-17/MAPK/mTOR/FoxO) and regeneration-associated Hippo/Wnt pathways. Based on these findings, we developed a novel dual-pathway small-molecule combination therapy C2, which simultaneously targets the hyperinflammatory response and impaired organ repair in sepsis. We validated that C2 significantly suppressed systemic and organ-specific inflammation, ameliorated multi-organ dysfunction, and improved survival in septic mice, and confirmed that its superior efficacy stems from the synergistic effect of dual-pathway co-targeting, rather than a single added component. Mechanistically, C2 reversed the dysregulation of core inflammatory and regenerative pathways, restored immune homeostasis, and normalized the aberrant miRNA-mRNA regulatory network identified in clinical sepsis patients. The C2 combination has favorable clinical translational potential due to its mechanism-driven design, patient-derived therapeutic targets, and clinically available small-molecule components. This study not only advances our understanding of sepsis pathogenesis, but also provides a promising, translatable therapeutic strategy for clinical sepsis management.

## Data Availability

The original contributions presented in the study are included in the article/**Supplementary Material**. Further inquiries can be directed to the corresponding authors.

## Supplementary material

Supplementary Table S1 RT-qPCR primers of mRNA.

Supplementary Table S2 RT-qPCR primers of miRNA.

Supplementary Table S3 The quantitative results of biochemical markers in **Figures 7B–E**.

Supplementary Table S4 The quantitative histopathological injury scores of the heart in **Figure 9A**.

Supplementary Table S5 The quantitative histopathological injury scores of the liver in **Figure 9A**.

Supplementary Table S6 The quantitative histopathological injury scores of the lung in **Figure 9A**.

Supplementary Table S7 The quantitative histopathological injury scores of the kidney in **Figure 9A**.

Supplementary Figure S1 The original, uncropped full membrane scans of **Figure 6G**.

Supplementary Figure S2 The original, uncropped full membrane scans of **Figure 8J**.

Supplementary Figure S3 The original, uncropped full membrane scans of **Figure 8K**.

Supplementary Figure S4 The original, uncropped full membrane scans of **Figure 8L**.

Supplementary Figure S5 The original, uncropped full membrane scans of **Figure 8M**.

Supplementary Figure S6 The original, uncropped full membrane scans of **Figure 10G**.

Supplementary Figure S7 The original, uncropped full membrane scans of **Figure 10H**.

Supplementary Figure S8 The original, uncropped full membrane scans of **Figure 10I**.

Supplementary Figure S9 Single-compound intervention validates the direct target regulatory effect of each compound in the LPS-induced A549 cell sepsis model. Relative mRNA expression of core genes of the multi-omics identified dysregulated pathways, including *Rela* **(A)**, *Stat3* **(B)**, *Mapk14* **(C)**, *Foxo3a* **(D)**, *Mtor* **(E)**, *Yap1* **(F)**, *Wwtr1* **(G)**, and *Ctnnb1* **(H)**.

Supplementary Figure S10 Safety and toxicity assessment of C2 combination in CLP-induced septic mice. **(A)**: Dynamic changes of body weight in mice from each group from the day before surgery (Day 0) to the 7th day after surgery (Day 7) during continuous daily administration. **(B)**: Daily general behavioral score of mice in each group, evaluated in a single-blinded manner according to standardized scoring criteria. **(C–F)**: Dynamic monitoring of serum liver and renal function biomarkers in mice from each group during the 7-day experimental period: ALT **(C)**, AST **(D)**, BUN **(E)**, CREA **(F)**. Statistical analysis: One-way ANOVA with Tukey’s *post-hoc* test **(B–F)**; n = 5 per group; Data are presented as the mean ± SD; **p* < 0.05, ***p* < 0.01, ****p* < 0.001, *****p* < 0.0001, ns, no significant difference.
