# Supplementary figures and images for: Integrated multi-omics deciphers sepsis immune dysregulation: a dual-pathway targeted small-molecule therapy improves survival and ameliorates multi-organ dysfunction
Source: Front Immunol. 2026 May 15;17:1809540. doi: 10.3389/fimmu.2026.1809540 (PMC13218904; doi:10.3389/fimmu.2026.1809540)

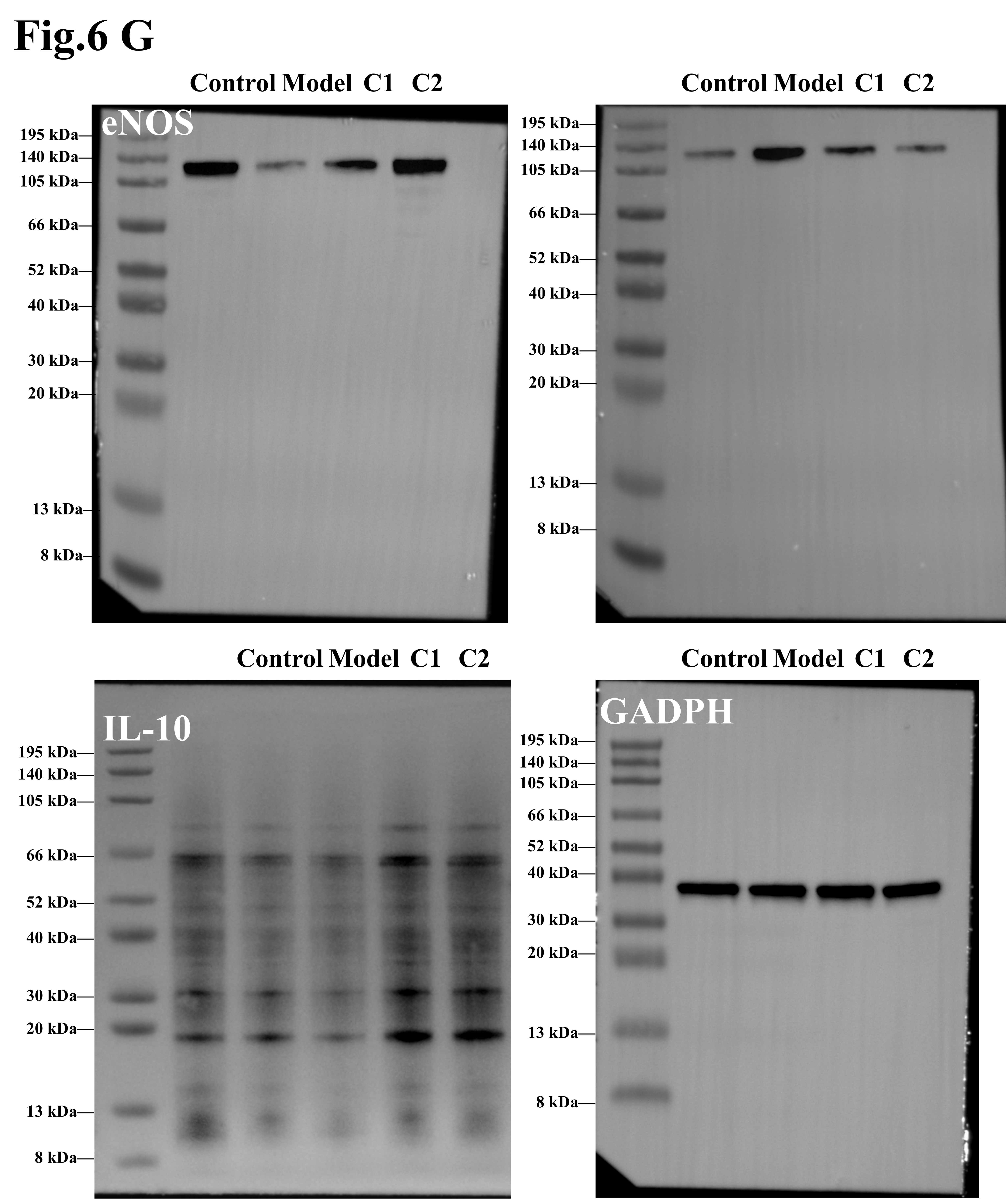

Supplement: Supplementary Figure S1 — The original, uncropped full membrane scans of Figure 6G. [file Image1.tif]

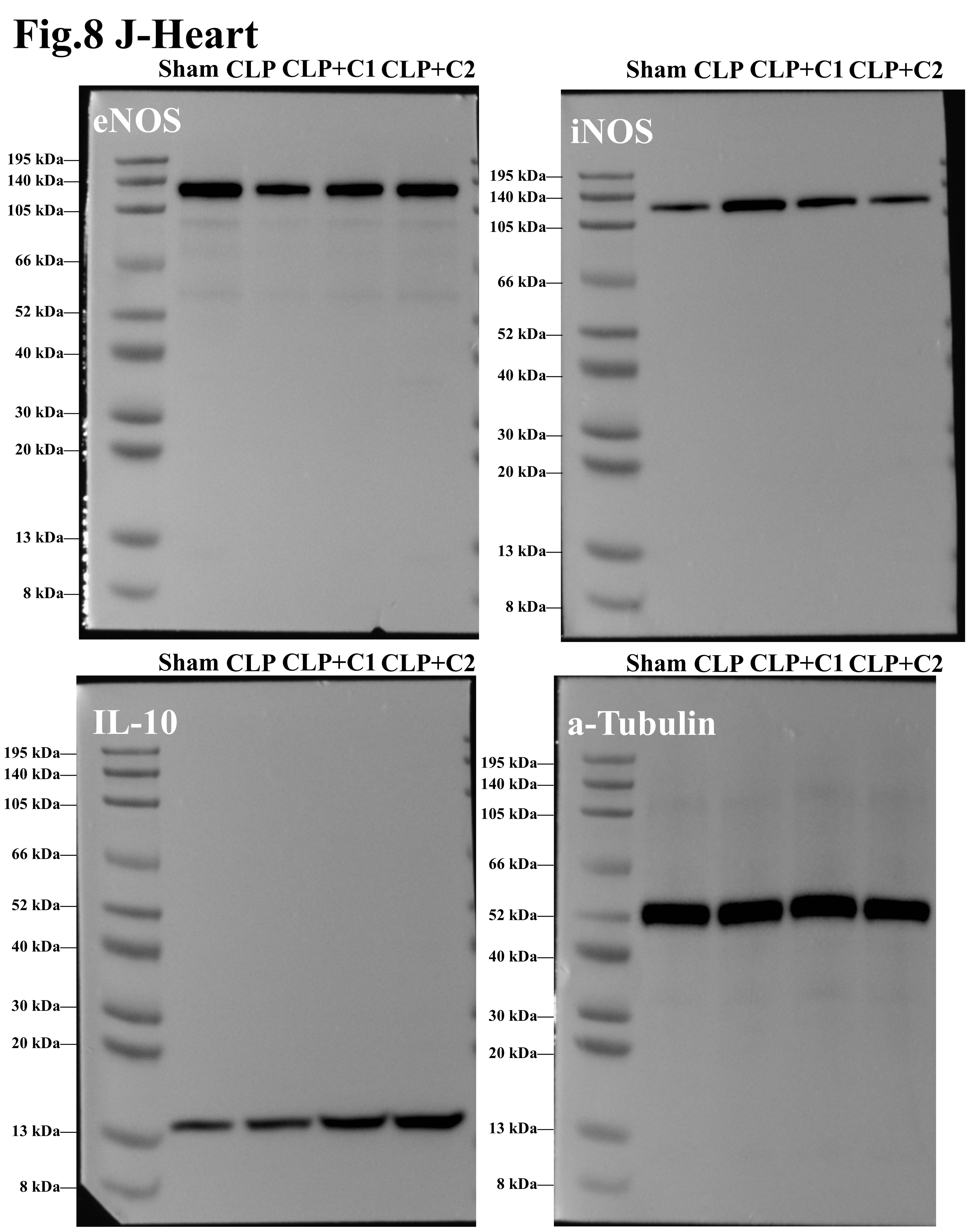

Supplement: Supplementary Figure S2 — The original, uncropped full membrane scans of Figure 8J. [file Image2.tif]

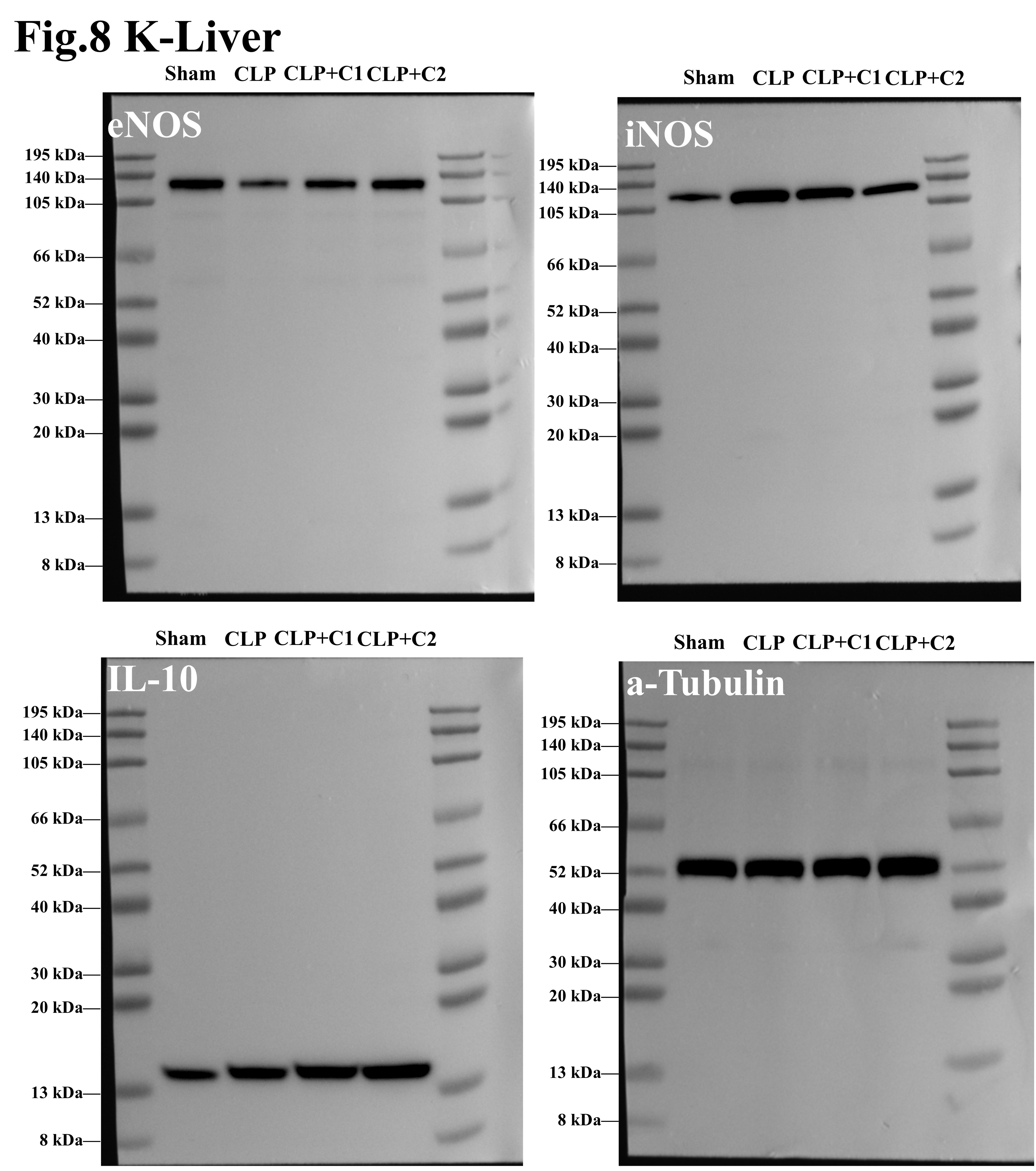

Supplement: Supplementary Figure S3 — The original, uncropped full membrane scans of Figure 8K. [file Image3.tif]

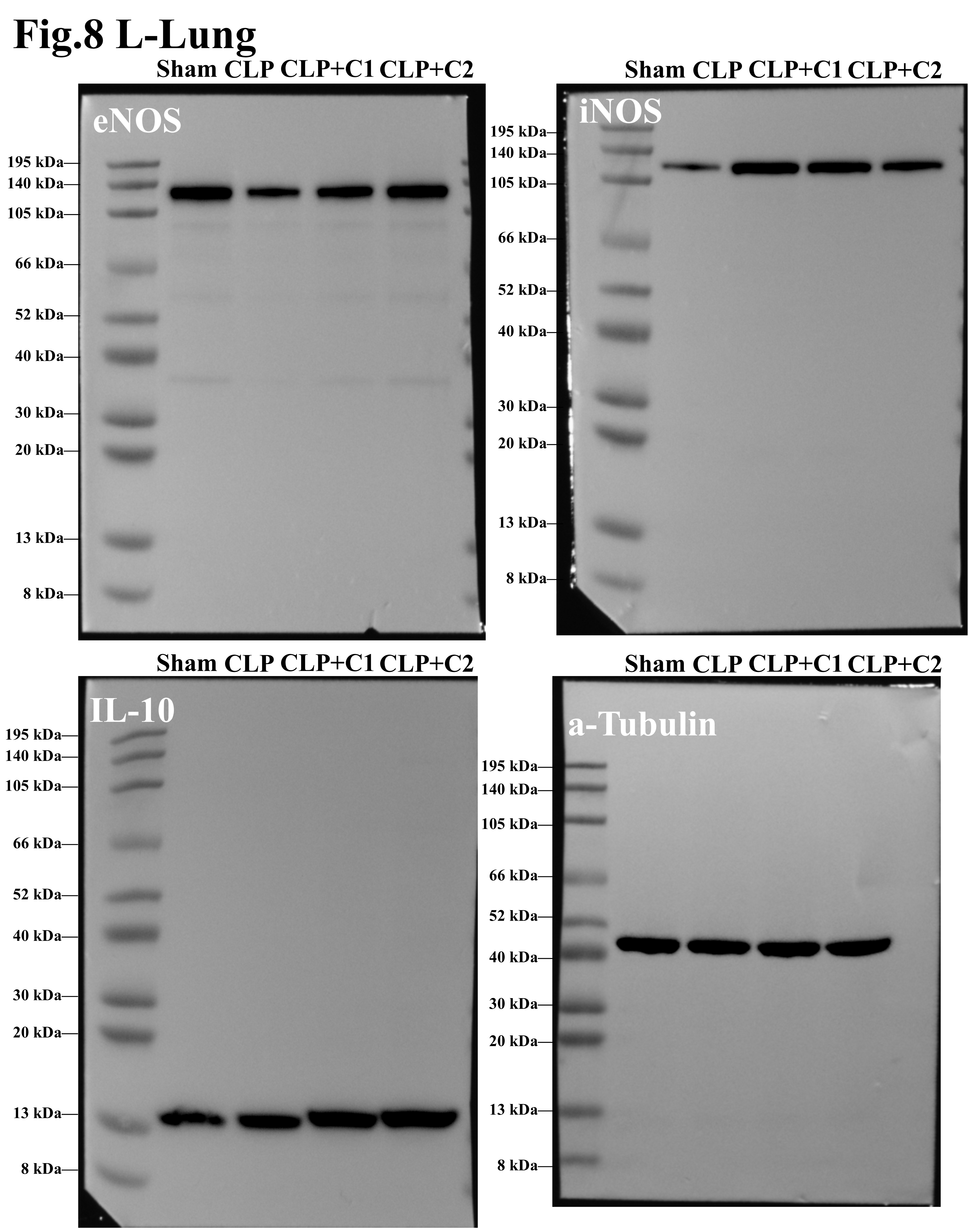

Supplement: Supplementary Figure S4 — The original, uncropped full membrane scans of Figure 8L. [file Image4.tif]

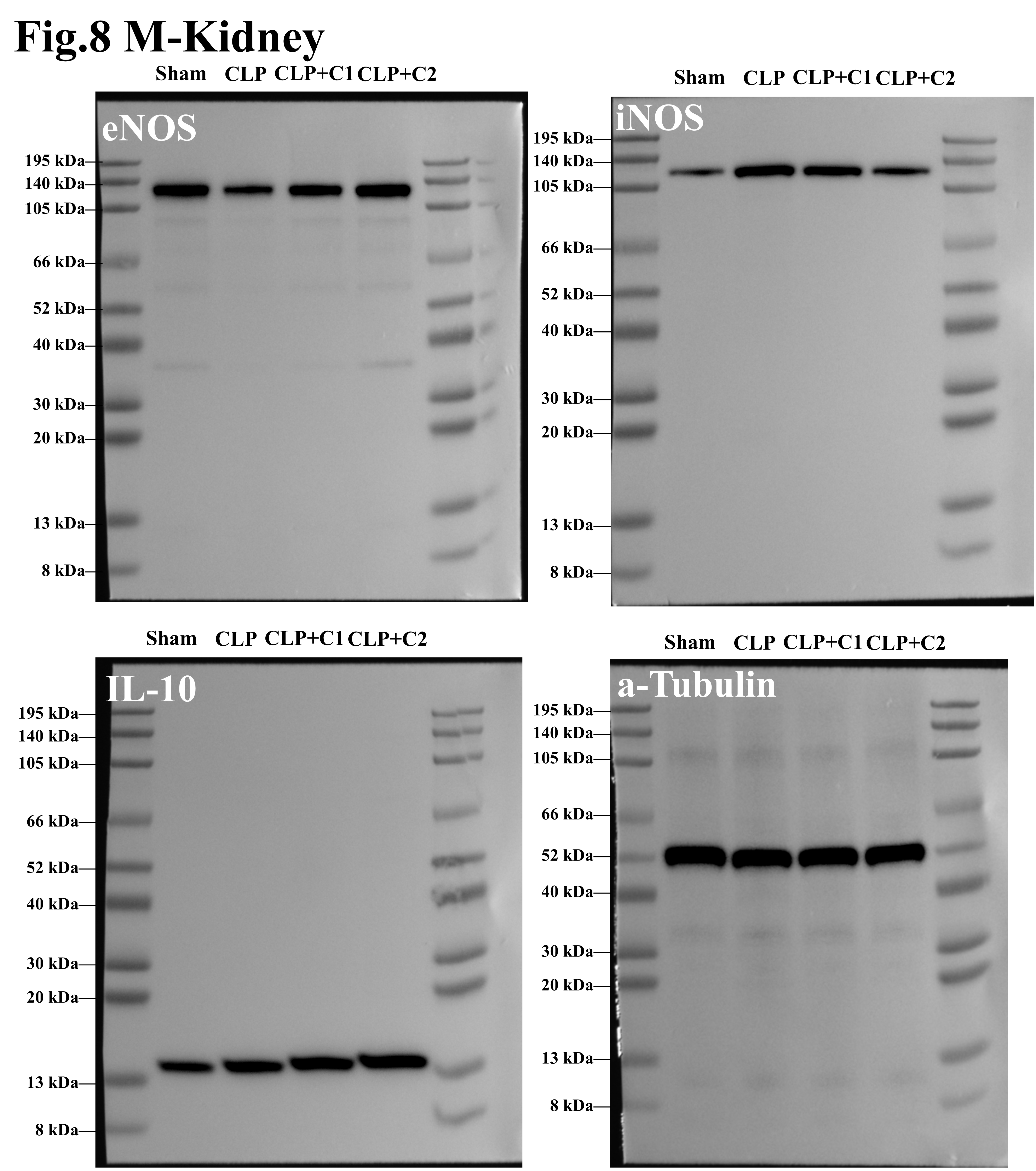

Supplement: Supplementary Figure S5 — The original, uncropped full membrane scans of Figure 8M. [file Image5.tif]

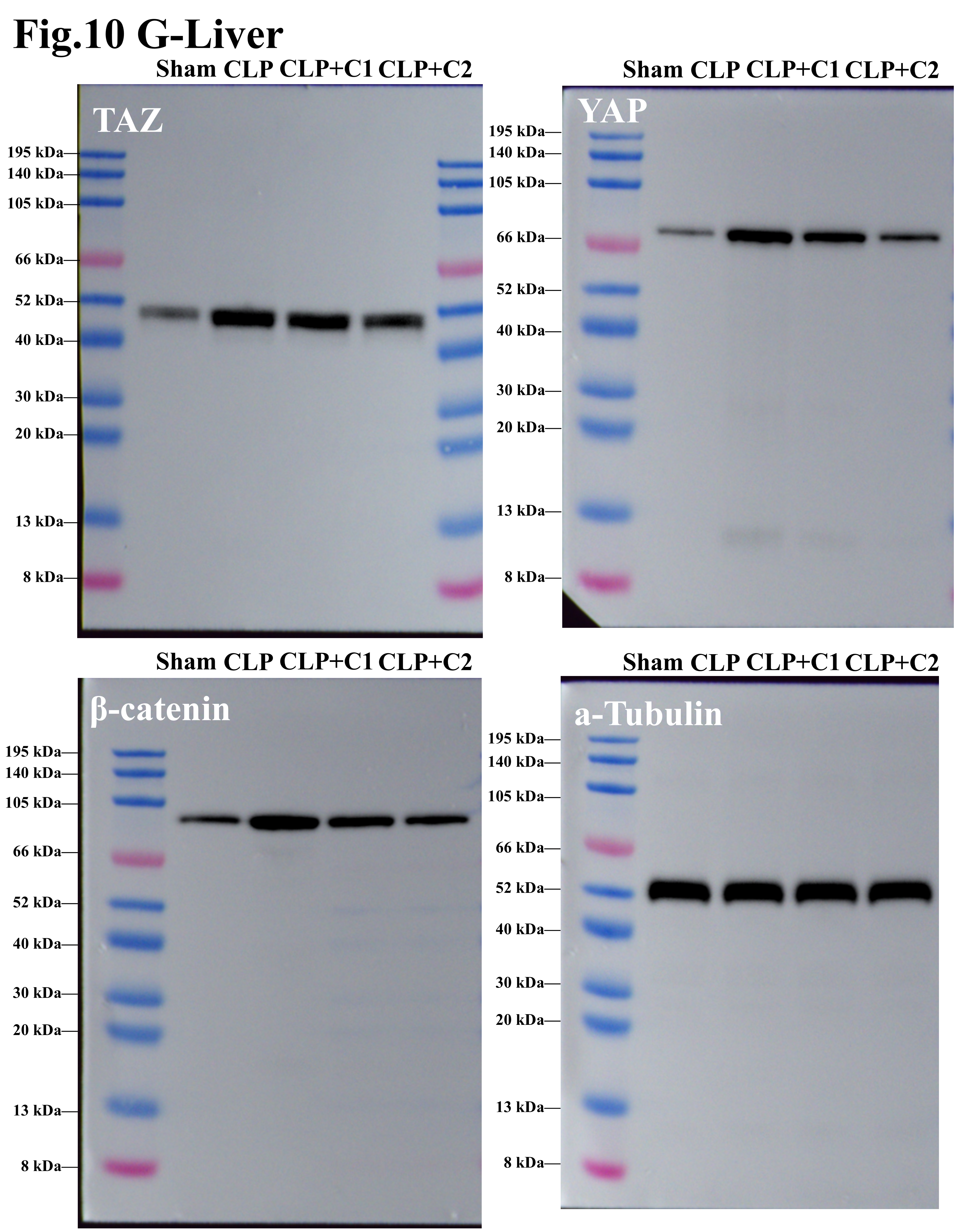

Supplement: Supplementary Figure S6 — The original, uncropped full membrane scans of Figure 10G. [file Image6.tif]

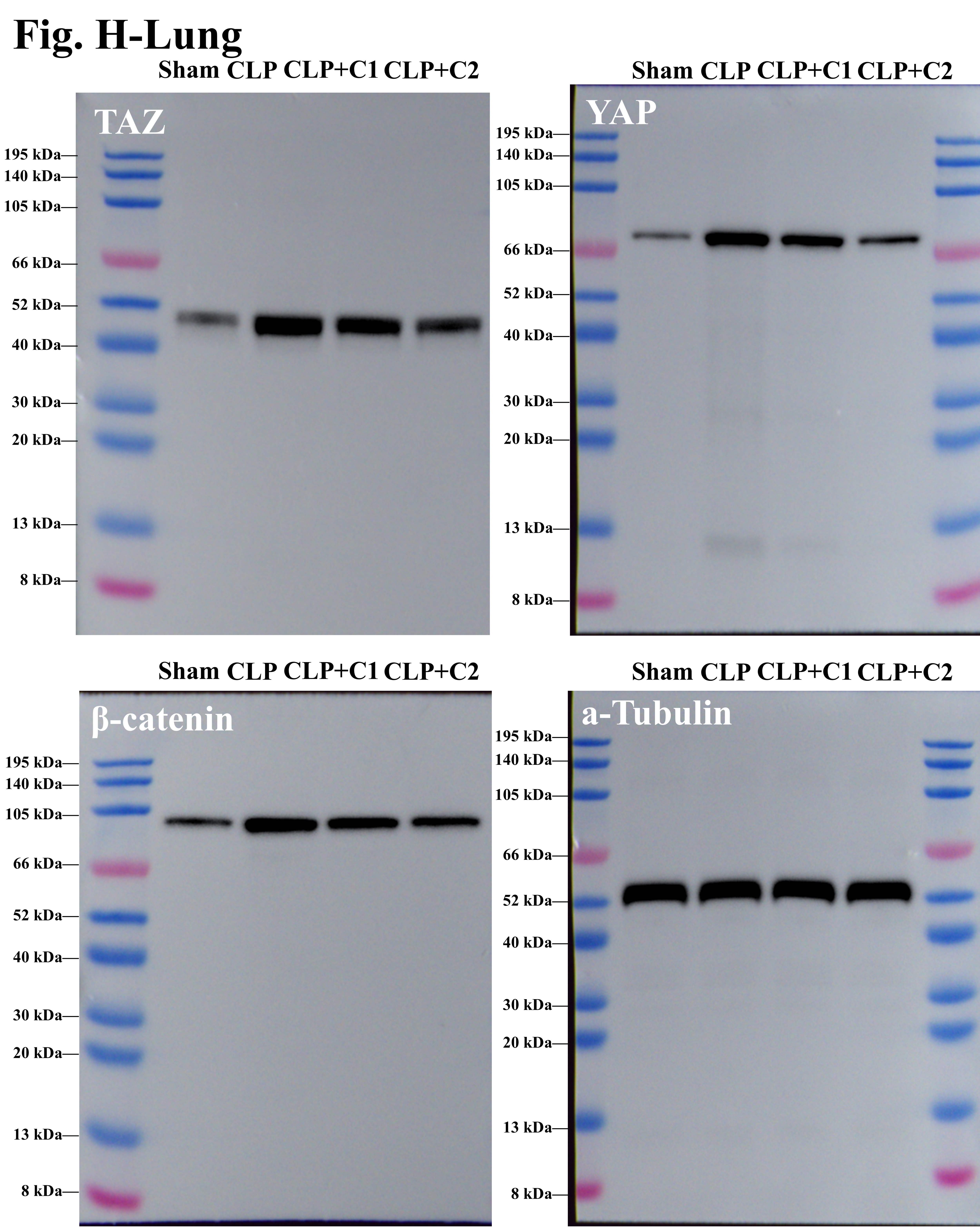

Supplement: Supplementary Figure S7 — The original, uncropped full membrane scans of Figure 10H. [file Image7.tif]

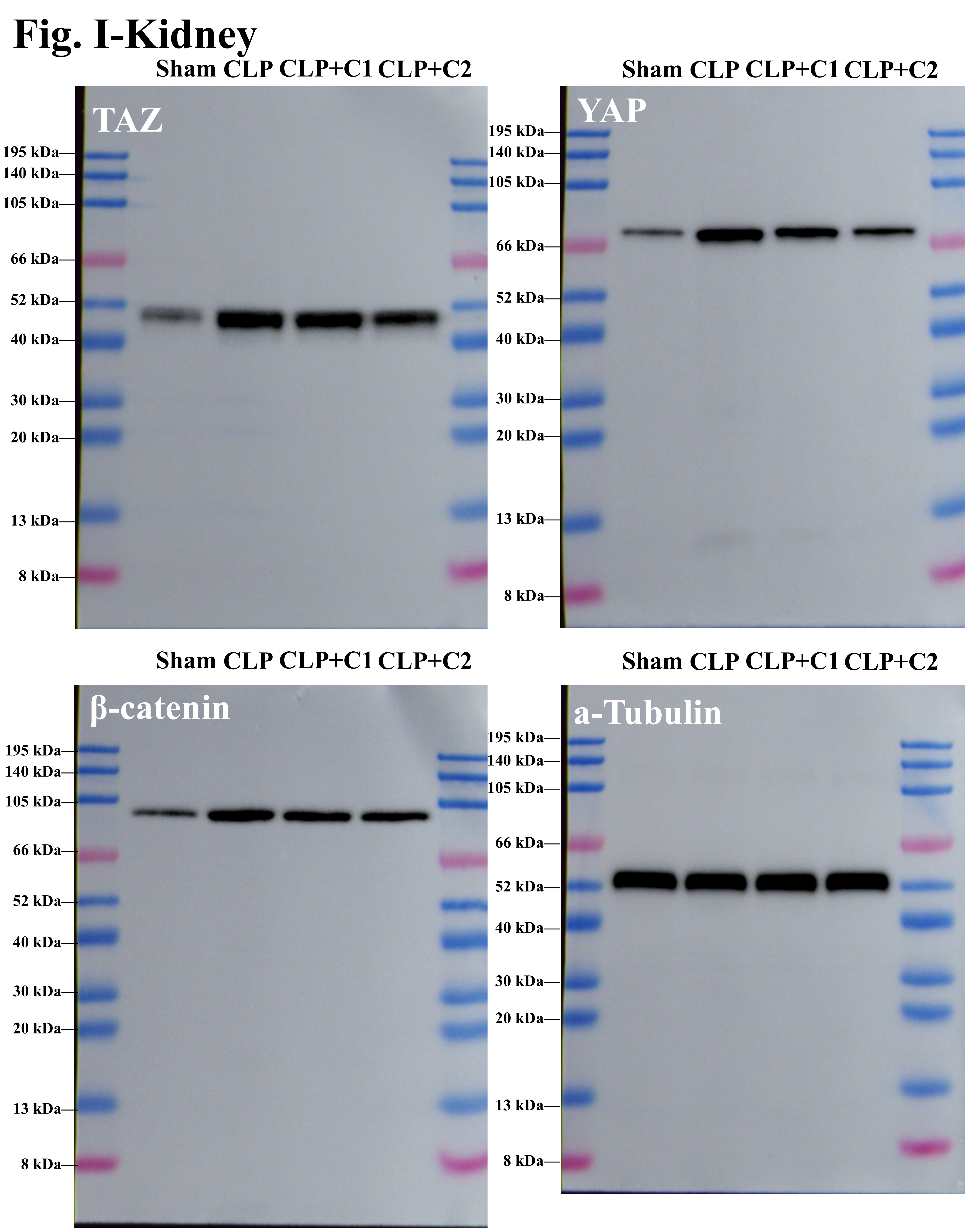

Supplement: Supplementary Figure S8 — The original, uncropped full membrane scans of Figure 10I. [file Image8.tif]

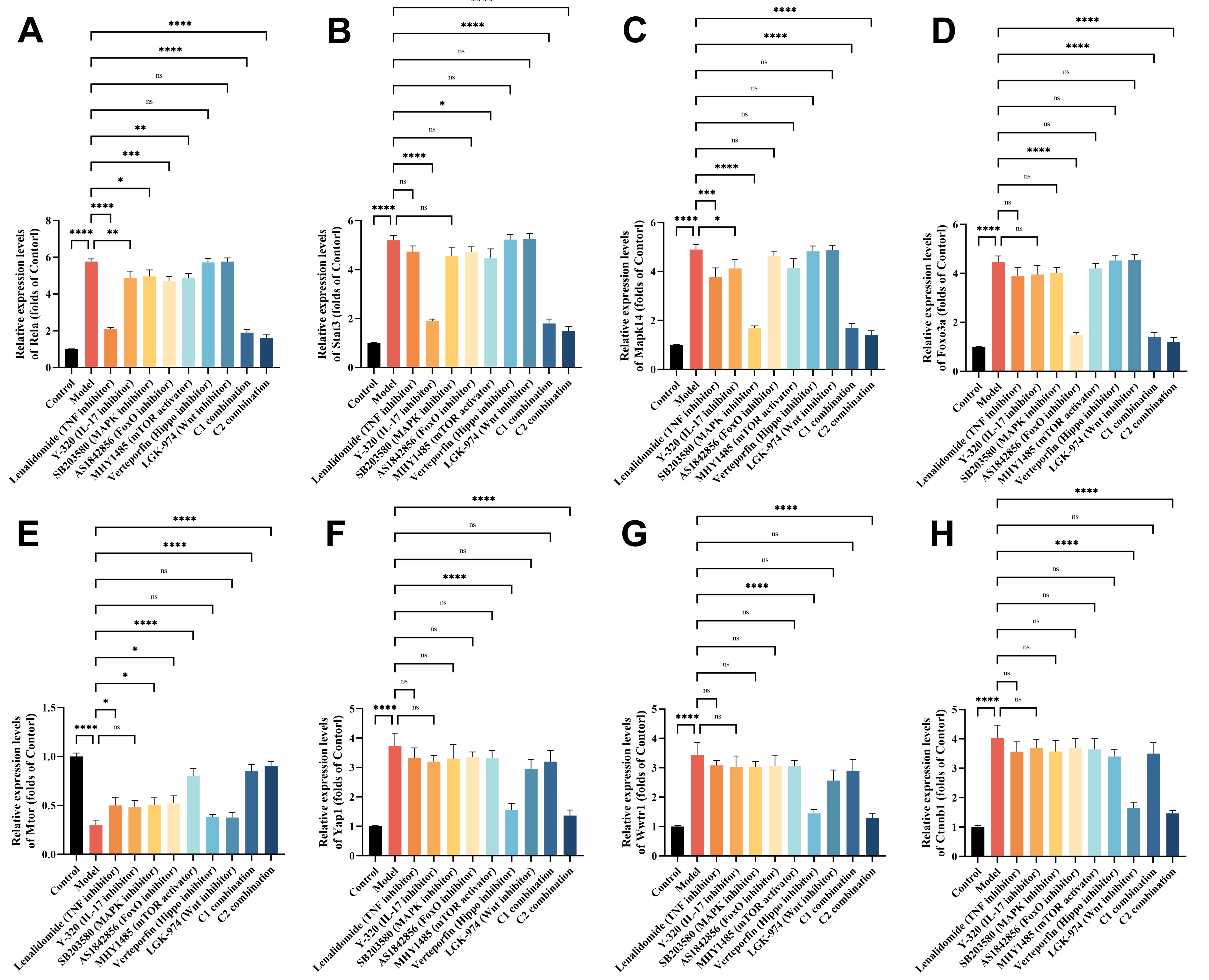

Supplement: Supplementary Figure S9 — Single-compound intervention validates the direct target regulatory effect of each compound in the LPS-induced A549 cell sepsis model. Relative mRNA expression of core genes of the multi-omics identified dysregulated pathways, including Rela (A), Stat3 (B), Mapk14 (C), Foxo3a (D), Mtor (E), Yap1 (F), Wwtr1 (G), and Ctnnb1 (H). [file Image9.tif]

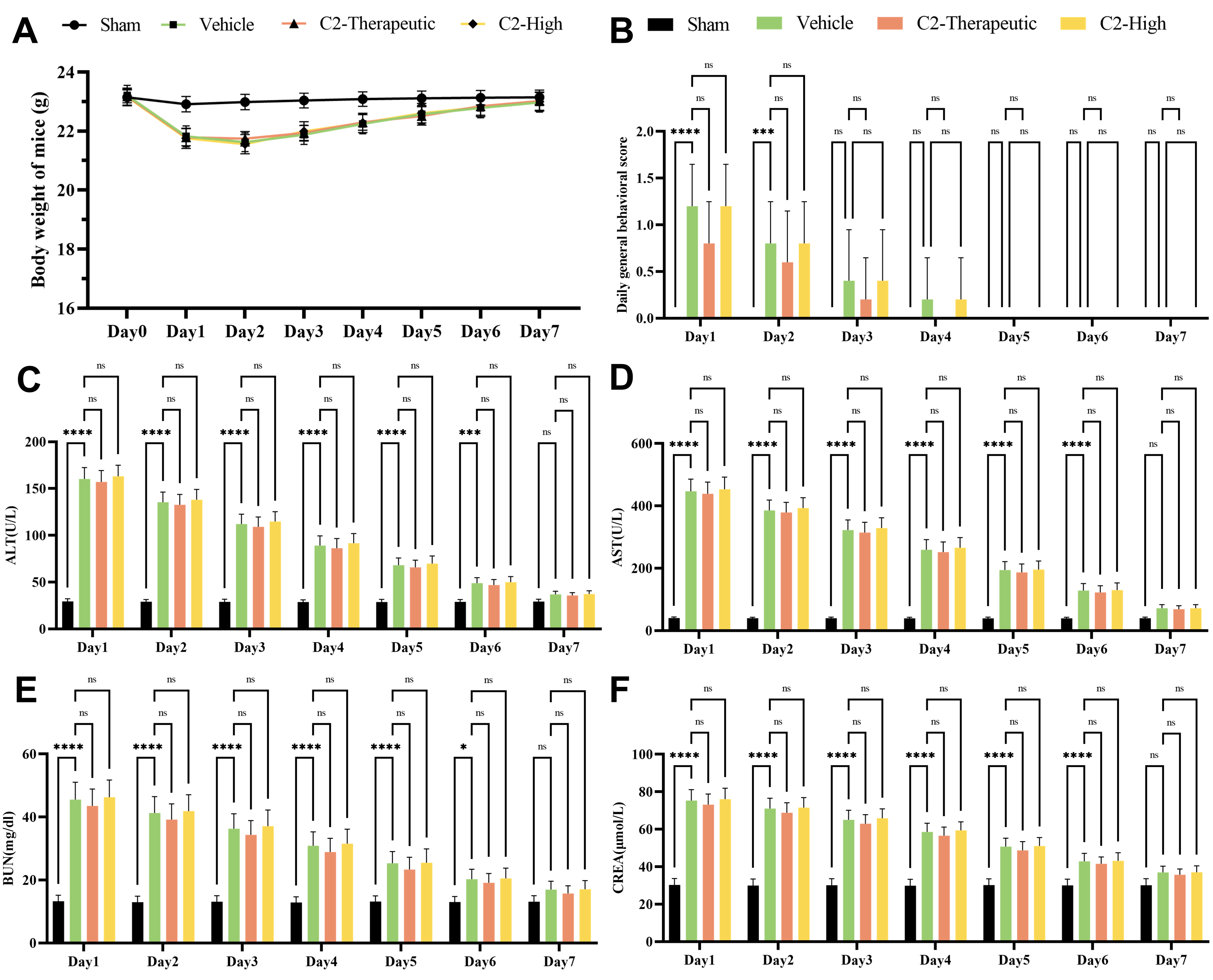

Supplement: Supplementary Figure S10 — Safety and toxicity assessment of C2 combination in CLP-induced septic mice. (A): Dynamic changes of body weight in mice from each group from the day before surgery (Day 0) to the 7th day after surgery (Day 7) during continuous daily administration. (B): Daily general behavioral score of mice in each group, evaluated in a single-blinded manner according to standardized scoring criteria. (C–F): Dynamic monitoring of serum liver and renal function biomarkers in mice from each group during the 7-day experimental period: ALT (C), AST (D), BUN (E), CREA (F). Statistical analysis: One-way ANOVA with Tukey’s post-hoc test (B–F); n = 5 per group; Data are presented as the mean ± SD; *p < 0.05, **p < 0.01, ***p < 0.001, ****p < 0.0001, ns, no significant difference. [file Image10.tif]
